# Interpersonal coordination in communication: effects of alignment in multiple modalities on objective and subjective task outcomes

**DOI:** 10.3389/fpsyg.2026.1655164

**Published:** 2026-02-25

**Authors:** Luca Béres, Péter Nagy, Tibor Pólya, Béla Weiss, Ádám Boncz, István Winkler

**Affiliations:** 1 HUN-REN Research Centre for Natural Sciences, Institute of Cognitive Neuroscience and Psychology, Budapest, Hungary; 2 Department of Cognitive Science, Faculty of Natural Sciences, Budapest University of Technology and Economics, Budapest, Hungary; 3 Department of Artificial Intelligence and Systems Engineering, Faculty of Electrical Engineering and Informatics, Budapest University of Technology and Economics, Budapest, Hungary; 4 Department of Social and Intercultural Psychology, Faculty of Social Sciences and Humanities, Károli Gáspár University of the Reformed Church in Hungary, Budapest, Hungary; 5 Brain Imaging Centre, HUN-REN Research Centre for Natural Sciences, Budapest, Hungary; 6 Machine Perception Research Laboratory, HUN-REN Institute for Computer Science and Control, Budapest, Hungary; 7 Hawkes Institute, Department of Computer Science, University College London, London, United Kingdom

**Keywords:** behavioral synchrony, communication efficacy, face-to-face communication, interpersonal coordination, verbal communication

## Abstract

**Introduction:**

Previous research has shown that during interactions, partners adapt to (imitate, synchronize, complement) each other’s behavior: a phenomenon often termed interpersonal coordination (IC). Approaches focusing on shared conceptual space suggested that the presence of synchronous or coordinated behaviors indicates the extent of conceptual alignment and thus, predicts communication success, while dynamical systems theory regards IC emerging from general coupling principles assuming no mechanistic role in the outcome of the interaction. Contrasting these two approaches, we tested whether IC appears in a wide variety of behaviors and how well various forms of IC predict the outcome of the interaction.

**Methods:**

Pairs of participants solved a computer-mediated communicative task involving verbal negotiation, while data of head motion, pupil size, and gaze direction were collected, and measures of prosody and structural speech characteristics were extracted from the recorded verbal interactions. Communication success was assessed using objective task performance measures and subjective evaluations from the participants.

**Results:**

(1) Interlocutors coordinated multiple aspects of their behavior, (2) some of the objective measures of task performance were predicted by gaze pattern coordination, and (3) some forms of IC were positively, while other forms of IC were negatively associated with the participants’ subjective experience of their partner and the interaction.

**Discussion:**

The results indicating that interpersonal coordination between interlocutors appears across multiple modalities are fully compatible with dynamical systems theory. On the other hand, the presence of both positive and negative associations between IC and subjective outcomes of the interaction suggests that while a strict form of a theory suggesting that stronger alignment leads to better communication outcome is not supported by the data, it is compatible with an extended version of such a theory that acknowledges the potentially different roles of partners in a joint task situation.

## 1 Introduction

Interpersonal coordination - referring to the temporal covariation of behaviors, physiology and cognitive/emotional states between individuals - is a ubiquitous feature in face-to-face interactions (daSilva and Wood, 2024). There is a growing body of evidence for the presence of interpersonal coordination under a number of different contexts (everyday conversations, musical ensembles, collaborative problem-solving, etc.) and across different modalities (behavioral, physiological, neural; for reviews, see Chartrand and Lakin, 2013; Ayache et al., 2021). It has been demonstrated that people spontaneously coordinate their speech characteristics (linguistic, syntactical choices, and prosodic features; Brennan and Clark, 1996; Branigan et al., 2007; Levitan and Hirschberg, 2011), postural sway (Shockley et al., 2003), head motion (Hale et al., 2020; Fujiwara and Daibo, 2016), and gaze direction (Richardson and Dale, 2005) when communicating face-to-face with each other. Some studies also show evidence for physiological synchrony concerning pupil size (Wohltjen and Wheatley, 2021), heart rate, and skin conductance (electrodermal activity or EDA; Behrens et al., 2020). Note that we refer to interpersonal coordination (henceforth IC) as an umbrella term which encompasses both mimicry (sequential alignment) and synchrony (concurrent or time-locked alignment) (Bernieri and Rosenthal, 1991), although in the literature these terms are sometimes used interchangeably (along with other terms such as convergence, coupling, entrainment, accommodation, etc.).

Although theoretical proposals about the putative role of IC in facilitating face-to-face communication have been put forth (Pickering and Garrod, 2013; Stolk et al., 2016), empirical evidence is still relatively limited. Interpersonal Alignment Theories (IAT, Pickering and Garrod, 2004, 2013; Stolk et al., 2016) argue that shared representations across individuals are essential for successful communication (to reach mutual understanding), because communicative actions, such as speech, facial displays, gestures, etc. tend to be ambiguous to the extent that their meaning depends on knowledge shared by the interlocutors. For example, the word “thing” or a simple grin can have many meanings depending on the context. In this context, mental representations are regarded as cognitive constructs, which can be examined at will and entered into cognitive operations (see, e.g., Fodor, 1975). Since shared concepts are not observable directly, the extent of shared conceptual knowledge between interlocutors is often inferred from synchronized/aligned behavioral programs or neural activity (Pickering and Garrod, 2004; Stephens et al., 2010; Stolk et al., 2014). According to IAT, mental alignment of situation models is a prerequisite for successful communication, which can be achieved through alignment percolating from lower levels of representations (phonology, syntax, and eventually semantics), ‘enhancing’ one another. Thus, IAT assumes links between behavioral alignment and mental alignment, and between mental alignment and communication success.

One may, however, question whether coordinated behavior or synchronized physiology are indeed closely related to shared conceptual knowledge between the communicating agents at all. The goal of Dynamical Systems Theory (DST) is to explain why IC emerges in interactions between living organisms. The core of this theory suggests that IC results from general coupling principles, which are not dependent on communicative intent (see Shockley et al., 2009; Dale et al., 2013 for coupling-based explanations of interactions, Paxton, 2023 for a review on DST, and Friston and Frith, 2015 for a predictive coding based approach of interpersonal synchronization). Theories under the umbrella of DST (which we refer to as a set of core concepts common to all variants of DST) usually describe the interaction partners as complex, self-organizing systems exerting physical constraints on each other and often consider IC as an emergent property of human (or non-human) interactions, without specifically relating these phenomena to alignment of conceptual/mental representations. The latter is also supported by the fact that motor coordination spontaneously emerges even in the absence of any goal or verbal interaction (Koul et al., 2023).

As a side effect of these theoretical distinctions, most studies investigating linguistic, prosodic alignment adapt the IAT approach (see, e.g., Nenkova et al., 2008), whereas IC of other behaviors such as movement are often discussed under DST (for example Fujiwara and Daibo, 2016; Paxton and Dale, 2017). One important consequence of the difference between IAT and DST is that IAT predicts a positive link between coordinated behavior/physiology and the outcome of communication (measured by the performance of a dyad in a communicative task), whereas DST makes no such prediction. It treats IC as an inherent feature of any interaction between two complex systems and provides a mechanistic explanation as to why IC arises. In this regard, DST is not particularly concerned with the outcome. However, these two approaches are not mutually exclusive (e.g., Hasson et al., 2012 who takes on a similar approach to DST but through additional considerations, also emphasizes the role of shared representations between interactants and how those might affect the outcome of an interaction). In addition, not all forms of IC are equal: different forms of IC might operate on different scales and might not be comparable to one another in their effect on communication success. Therefore, assessing which forms of IC best predict the outcomes of a conversation may shed light on the underlying theoretical questions. Here we will survey the literature with this question in mind.

An important part of the evidence supporting the effects of IC on communication outcome comes from the field of psycholinguistics. Structural and linguistic alignment in speech appears to improve mutual understanding in conversation (Ferreira et al., 2012), performance on a communicative computer game, and the perceived naturalness and flow of conversation (Nenkova et al., 2008). Further, acoustic/prosodic alignment has been associated with communication success on a perceptual task (Borrie et al., 2015) and the ‘naturalness’ of the conversation (De Looze et al., 2014). It is important to note, however, that more linguistic alignment is not necessarily better, as there is evidence that task constraints modulate the level and specificity of alignment needed for successful communication (as measured by task performance; Fusaroli et al., 2012).

Other forms of coordination have also been found to show a positive relationship with communication outcome in a task-related manner. For example, Richardson and Dale (2005) found that the coordination of gaze direction between speaker and listener during a storytelling monologue resulted in better comprehension, measured by recall tests. Another study looked at how programmers use gaze direction to guide their attention to specific parts of a code during a joint programming task involving verbal communication (Jermann and Nüssli, 2012). The authors found that pairs who had higher cross-recurrence of gaze patterns (or gaze coordination) have shown higher interaction quality, which was determined by various aspects of problem-solving, such as agreeing on strategy, division of labor, participation symmetry, etc. IC of gaze patterns is strongly dependent on the task at hand. For example, in a joint visual search task, where participants had to find the differences between two images, gaze coordination was lowest when they could freely communicate with each other verbally as opposed to when they were only allowed to provide backchannel feedback (like “yes,” “hm”) or could not talk at all (Coco et al., 2018). In the no verbal interaction condition, gaze coordination was found to have a negative effect on task performance, whereas in the full dialogue condition no effect was found. However, in this particular task, the wider spatial distribution of gaze (essentially less alignment) among the participants allowed for faster scanning of the visual space, which in turn led to higher task success.

IC in motor behavior is prevalent during communicative interactions and has been found to have an effect on communication outcome. For example, following synchronous motion individual performance of dyad members was enhanced in a subsequent communicative problem-solving game compared to members of dyads, who were instructed to move asynchronously beforehand (Miles et al., 2017). Note, however, that joint performance did not significantly improve in this study. Another study by Tsuchiya et al. (2020) showed that IC measured with motion sensors correlated with the amount of information that was exchanged between participants, although the authors acknowledge that their dataset consisted of only 9 dyads, which limits the robustness of the results. It has also been demonstrated that under adverse conditions, such as noisy environment and increase in task difficulty, the IC of motor behavior increases (Hadley and Ward, 2021), and although it is not direct evidence for the effect of IC on communication outcome per se, it is consistent with the notion that IC facilitates communication.

The level of IC between interacting persons (assessed by various measures) is also affected by contextual variables. In order to assess whether such variables then have a similar effect on the success of communication, selected contextual variables were manipulated in the current study. Regarding all forms of IC, IC is thought to increase with the level of familiarity and liking (McIntosh, 2006; Latif et al., 2014), interaction quality (Julien et al., 2000), and rapport (Miles et al., 2010), whereas it decreases in argumentative situations (Paxton and Dale, 2013, 2017). Increasing the difficulty of the task—either through perturbation to the vocal channel (Hadley and Ward, 2021) or through limited availability of information to the interlocutors (Louwerse et al., 2012)—has been also shown to increase the level of movement coordination between communicating agents. There is also evidence that the shared knowledge between participants about a given subject positively influences the level of gaze coordination between them during a subsequent conversation related to that particular subject (Richardson et al., 2007a). Another important aspect of IC is how it is modulated depending on the multimodal versus unimodal nature of the conversation. Multimodal information facilitates the emergence of coordination between participants compared to unimodal setups (see for example Dias and Rosenblum, 2016; Wright et al., 2014), while speakers compensate the removal of a modality by stronger alignment to their partners (see Placiński and Żywiczyński, 2023 for text-based versus face-to-face interactions, or Zhao et al., 2023 for alignment of facial expression in verbal versus non-verbal communication). Therefore, multimodality has no unambiguous effect on IC, as the effect depends on the context.

### 1.1 The current study

In the present study, we took a multimodal perspective for studying IC (as was recently suggested by Gordon et al., 2024): we designed an experiment where the level of coordinated behavior and physiology can be assessed during unconstrained on-line face-to-face conversation and their influence on the success of communication can be tested. We recorded speech (to estimate coordination in prosody and speech characteristics), gaze direction (to assess gaze coordination), pupil size (to assess pupil size coordination), and upper-body motion (to calculate motion coordination) simultaneously from two participants, while they solved a communicative task involving negotiations. Furthermore, we tested the effects of task difficulty, familiarity between the interlocutors, and unimodal versus multimodal communication.

For the purpose of operationalizing IC, we employed the integrative framework coined by Rasenberg et al. (2020) who argue that when quantifying IC, one has to consider the time, sequence, meaning, form, and modality dimensions of IC. On this basis, we considered IC as the alignment of behaviors (1) in an instantaneous or sequential manner over short temporal distances, (2) occurring within a sequence (of a given time window), (3) irrespective of whether the matching behavior has the same or different potential meaning or even any meaning (e.g., a scratch on the head can happen as matching behavior without specific meaning), (4) occurring with a similarity of form (with the exception of movement coordination where the covariation of motion energy was used as a measure of IC, for methodological details see Section 2.5.2), and (5) within the same modality (we do not investigate cross-modal alignment, i.e., alignment between gestures and words).

In the IAT framework, communication is successful if mutual understanding has been achieved. However, mutual understanding - similarly to mental representations - is a latent variable that cannot be directly measured. Rather, it must be inferred from the observable behavior. Here we employed objective measures of performance in a joint task as proxies of mutual understanding. Interaction between the partners within the joint task has also other consequences, such as changing their knowledge of and attitudes towards each other as well as towards the task, etc. To assess these results of the interaction, we also collected subjective measures: participant ratings about the conversation and their relationship to their partner, as these have also been shown to influenced by IC (e.g., Mayo et al., 2021; Ramseyer and Tschacher, 2011; Sharon-David et al., 2019). The objective and the subjective measures together form our assessment of the outcome of the communication during the task.

Based on the previously accumulated evidence on the pervasiveness of IC in communication we first and foremost hypothesized that IC of speech (prosodic, structural), gaze direction, pupil size, and head movement will spontaneously emerge during unconstrained task-oriented conversations (daSilva and Wood, 2024) [H1]. With respect to the theories brought up to explain face-to-face communication, we hypothesized that stronger IC (especially those most often investigated under IAT, such as prosodic and structural alignment of speech) will result in better communication outcomes: greater communication success (indexed by task performance; e.g., Borrie et al., 2015; Tsuchiya et al., 2020) and more favorable relationship outcomes between the interlocutors (indexed by subjective evaluations from the participants; e.g., Lakin and Chartrand, 2003; Ramseyer and Tschacher, 2011) [H2]. This test is important for finding support for IAT, whereas DST does not associate the strength of IC with the quality of the outcome of the interaction. Finally, we tested whether levels of IC distinguish between the levels of familiarity (unfamiliar or familiar), levels of task difficulty, and whether visual information was available or not. While for familiarity, the literature suggests a positive effect (e.g., McIntosh, 2006; Latif et al., 2014), for multi-modality (see Wright et al., 2014; Zhao et al., 2023) and task difficulty (Louwerse et al., 2012; Hadley and Ward, 2021), the effects observed proved to be context dependent. Here we assessed how the current manipulations affected IC.

## 2 Materials and methods

### 2.1 Participants

Data were collected from 124 adult same-gender pairs (248 individuals - see power analysis in Supplementary material, age: M = 21.4 years, SD = 2.7 years, 178 females, 24 left-handed) in three conditions conducted on three different groups of participants: the *Baseline condition*, the *Unfamiliar condition*, and the *Unimodal condition*. Demographic data of the three conditions are presented in Table 1. See section *2.2.3 Manipulating familiarity and modality of the communication* for the description of the conditions.

**Table 1:** Demographic data of the experimental conditions.

Demographics	Baseline condition	Unfamiliar condition	Unimodal condition
Number of pairs	40	43	41
Age (years)	Range = 18–32^a^ M = 21.4, SD = 2.6	Range = 18–38^b^ M = 21.4, SD = 3.2	Range = 18–28 M = 21.4, SD = 2.2
Female pairs	30	30	29
Left-handed individuals	6	9	9

^a^All but one participant fell into the 18–30 year age range. ^b^All but two participants fell into the 18–29 year age range.

Participants were randomly assigned to conditions and each pair participated in exactly one condition. Participants did not know each other prior to the study. They were recruited either from university courses or through student employment agencies. In case of university courses, participation was compensated by credit points. In case of student employment agencies, participation was financially compensated (approx. 6 euros per hour). Participants gave written informed consent after the aims and features of the experiment were explained to them. The study followed the Declaration of Helsinki guidelines. Ethical approval was granted by the local review board of the Institute of Cognitive Neuroscience and Psychology of the HUN-REN Research Centre for Natural Science (United Ethical Review Committee for Research in Psychology; EPKEB). All participants had normal or corrected-to-normal vision and normal hearing. None of the participants reported any psychological or neurological disorders.

### 2.2 Procedure

The data analyzed in this study were collected as part of a larger study designed to investigate the behavioral and neural dynamics of human face-to-face communication. In all three conditions, pairs of participants performed five main tasks in the following order: an introductory conversation (“Familiarization Phase”), a 2–5 rounds of a computer-mediated communicative game (“Bargaining Game” [BG]), a 15-min unstructured conversation (“Free Conversation”), subjective evaluation of the free conversation, and three short personality questionnaires. Data from the last three tasks and the EEG recording were collected for later analyses and, therefore, they will not be explained in detail here. The current study focused on data collected during the BG where participants were engaged in on-line (computer-mediated) face-to-face interaction. Following each round of BG and after the Free Conversation task, participants were asked to rate a few statements about their subjective experience of the previous conversation/task (e.g., smoothness of conversation, enjoyment of the task) and their partner (e.g., liking, feeling ‘in-sync’). Only questionnaires regarding the BG rounds will be detailed here. The experiment started with the “Familiarization Phase,” which was a 15-min free conversation between participants, designed to get them acquainted (participants received a few suggested topics, but were not specifically instructed to talk about them). In one of the experimental conditions (termed *Unfamiliar* in the text) participants talked with an experimenter instead of their partner, which was part of the familiarity manipulation (see *2.2.1 Manipulating familiarity and modality of the communication*). After the “Familiarization Phase,” participants were set up with sensors (see *2.3.1 Materials*) and the sound intensity was set at a comfortable level, separately for the two participants. Next, the rules of the BG were explained to each participant, based on a prewritten script, separately by one of the experimenters. Then, participants played a test run of the BG to familiarize themselves with the game. This test run also enabled the experimenters to check whether both participants understood the rules of the game. After the test run, participants played 2–5 rounds of the BG (depending on the time required to set up all sensors, and the duration of the games). The total duration of the experiment (including the preparation, the actual measurement, and the closing administration after the measurement) was 4 h, within which the BG took approximately 1 h (including the breaks between rounds).

#### 2.2.1 Manipulating familiarity and modality of the communication

Three different conditions were implemented with each pair participating in only one condition. In the Baseline condition, the two participants had a 15-min introductory conversation with each other (“Familiarization Phase”), and interacted multimodally (could see each other during the game through the camera stream). The Unfamiliar condition differed from the Baseline condition only in that the two participants did not meet before the BG, instead, they both had a 15-min conversation with one of the experimenters (similar to the conversation in the “Familiarization Phase”). Otherwise, everything was kept the same. The Unimodal condition differed from the Baseline condition only in that participants could not see each other through the camera during the game, instead, they saw a static picture of each other (the picture was taken before start of the BG by webcam; participants were instructed to show a neutral but friendly face). The three conditions along with the multiple rounds of BG enabled experimental manipulation along two dimensions: pre-task familiarity of the interlocutors and modality of communication (unimodal or multimodal).

#### 2.2.2 The bargaining game (BG)

The Bargaining Game (Galinsky and Mussweiler, 2001; Valley et al., 2002) was implemented in-house with Psychtoolbox (version 3.0.17) (Brainard and Vision, 1997; Pelli, 1997; Kleiner et al., 2007) in Octave (version 5.2.0) (Eaton et al., 1997, 2020). The BG was selected for the current study as it provides a good trade-off between everyday context and experimental control over communication outcome (measured by task performance). Everyday conversations with coworkers or friends often involve the process of coming to terms on certain matters and arriving at joint decisions - a cornerstone of cooperation. The BG models these situations well, as it creates a scenario where both parties can be successful at the same time (i.e., achieving their main task as well as by accumulating wealth). Overall success of the pair can then be assessed by the time it took to complete the main task of both partners and the joint wealth gain they reached during the game. The former will be used as a proxy of “communication efficacy,” because it reflects how fast different pairs of participants solved the same task. The latter is used as a proxy of “communication gain,” assessing the amount of profit participants realized together in the game. In addition, the BG also made it possible to investigate (a) symmetries in individual contributions of the partners, which is indexed by the difference measures (see *2.4.1 Task performance in the bg/measures of communication gain and efficacy*).

In the BG, both interlocutors had a few (8–8) different tokens (sellable items shown by icons on the display), only seeing information (quantity and price) of their own. They were instructed to verbally negotiate the trades for these tokens. Each participant was instructed to collect 3 different types of tokens with a specified minimum quantity (henceforth: “must-have” tokens), the type and amount only known by him/her. Participants were explained that they had the same token types but their pricing might be different (e.g., for participant A, the value of a “trash bin” could have been 30 arbitrary units, while for participant B, the same token could have had a value of 10). Their secondary task (beyond collecting the must-have tokens) was to increase their wealth and they could always see their own total wealth on the display (sum of prices of all tokens currently in their possession). The different pricing of tokens allowed creating non-zero-sum situations in which both partners could be successful in both tasks at the same time and they had equal chance for increasing their wealth.

**Figure 1:**
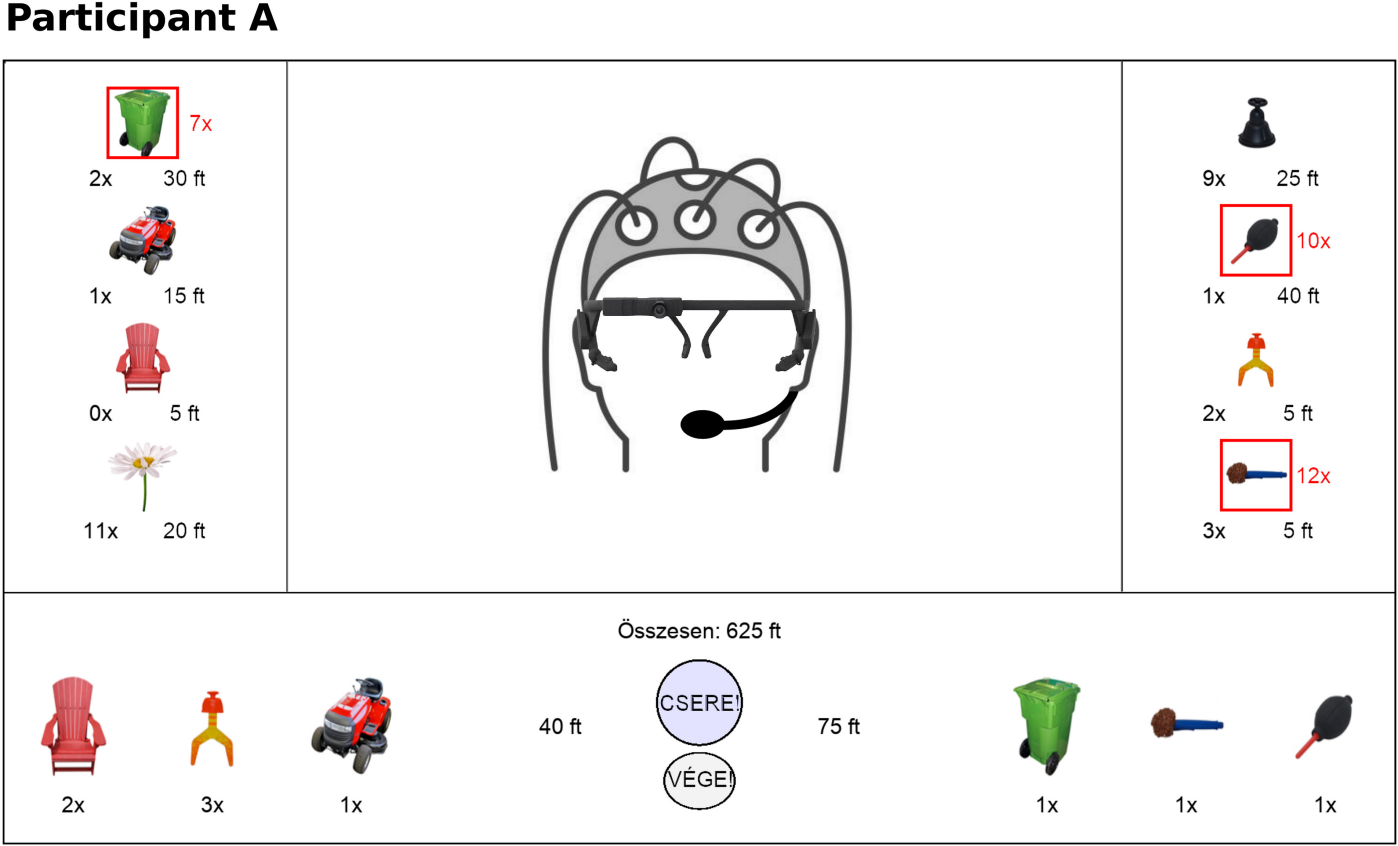
Graphical user interface (GUI) of the BG. The video (or static picture) of the other participant’s head was displayed in the center of the screen. On the sides, tokens available to the participant were shown, together with their prices in arbitrary units (ft) and quantities. Must-have tokens were marked with a red square and the required quantity was shown in red as well. The bottom of the display showed the area for tokens the participant marks to be sold (left) and bought (right) in a transaction, two buttons for exchange (upper button) and ending the game (lower button), and the total wealth (above the exchange button). An exchange happened when both players activated the exchange button (mutual agreement). For an illustration showing the screen of both participants at the same time (see Supplementary Figure 1).

Participants were seated in a shielded, sound-attenuated laboratory, in front of a computer screen, and controlled the game through a computer mouse. The screen displayed the on-line view of a camera showing the other participant’s head and the user interface of the BG (Figure 1; Supplementary Figure 1). In the Unimodal condition, the video of the other participant was exchanged for a static picture of him/her. Each round of the BG was limited to 15 min, but pairs were allowed to finish it earlier if both players collected the required minimum number of must-have tokens and they agreed that they cannot further increase their wealth in the current round. The number of exchanges was not limited. Participants were not able to see the token quantities, prices, or the total wealth of the partner’s side, however, they were allowed to share any information with their partner during the game if they wanted (e.g., prices, quantity of must-have tokens, etc.).

#### 2.2.3 Increasing the difficulty of the bargaining game

In all conditions, the difficulty of the task was increased from round to round in order to (a) test the effects of task difficulty on communication between the interlocutors (assessed similarly to the external manipulations of the different conditions), (b) maintain the richness of communication throughout the repeating task by preventing interlocutors from utilizing some learned quick solution in completing the task, and (c) maintain the interlocutors interest in the task. The interlocutors played 2–5 rounds of the game. In each round, new tokens were introduced to keep the participants engaged. Pictures for the tokens were chosen from the BOSS (Bank of Standardized Stimuli) database (Brodeur et al., 2010) and the NOUN (Novel Object and Unusual Name) database (Horst and Hout, 2016). The latter contains pictures of novel abstract tokens that do not have a specific name or function. Difficulty was increased between successive BG rounds by two factors: (1) the potential wealth flow (the potential increase in aggregated wealth of the two players if each token is transferred to the player who had the higher price for it) and (2) the number of different abstract tokens (taken from the NOUN database) included in each partner’s starting set. For a more detailed description (see Supplementary Table 1). Because only a few pairs reached the 5th round of the BG (26 pairs), only data from rounds 1–4 were analyzed.

### 2.3 Data acquisition

All data were recorded by computers with a common time-stamping system implemented through Network Time Protocol on the local-network (~1 ms precision) for collecting them into a coherent data set of continuous measures, which were then supplemented by demographical, evaluation, and psychological test data.

#### 2.3.1 Materials

**Figure 2:**
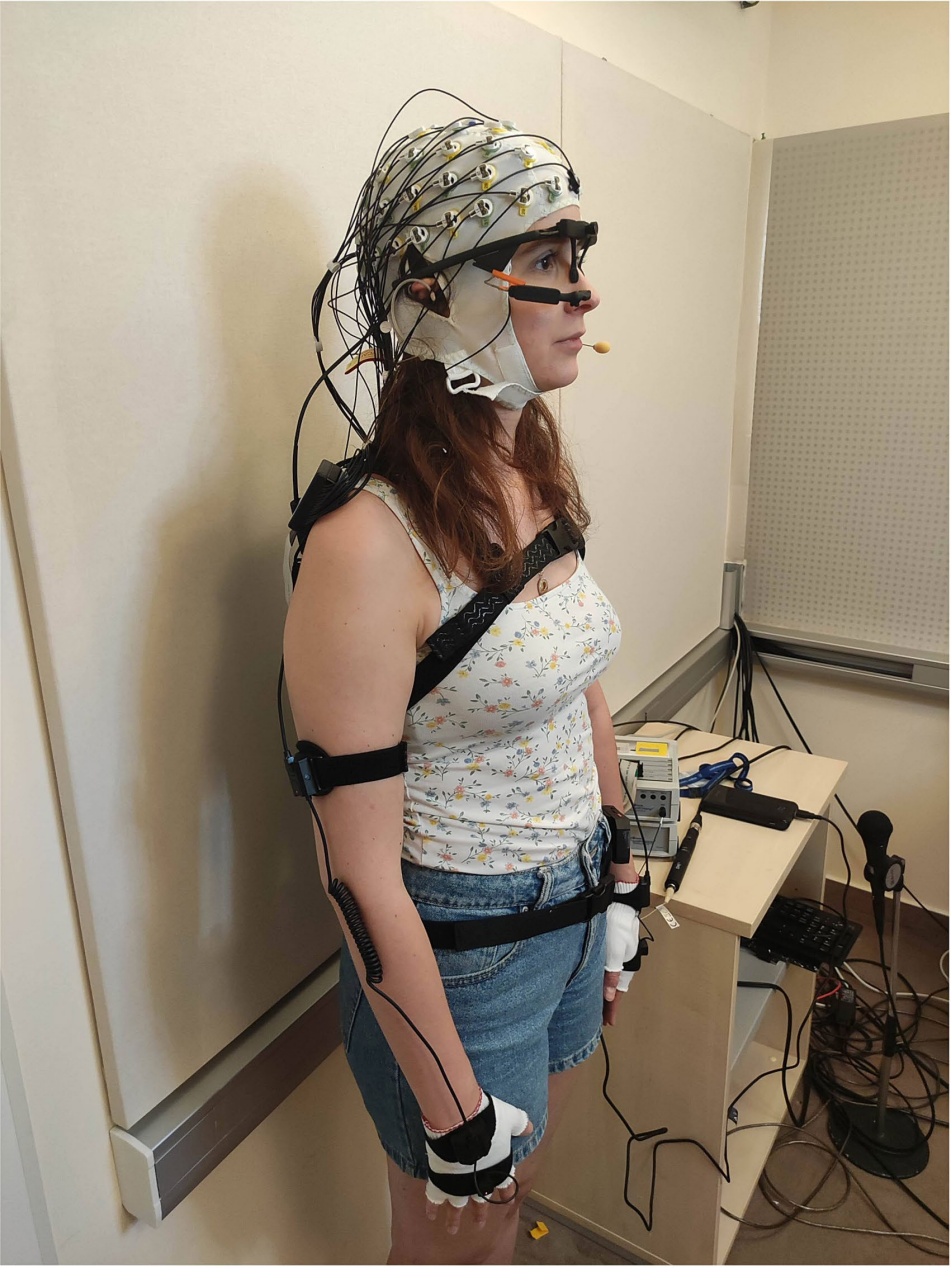
Sensors used in the BG. The participants were equipped with high-density EEG, wearable eye tracker, inertial motion capture system, and headset microphone.

Participants were equipped with high-density EEG, wearable eye tracker, inertial motion capture system, and a headset microphone (Figure 2). Speech was captured by the headset microphone and played back to the partner from a loudspeaker in the other laboratory. Video of the participant’s head was captured by a webcam and shown on-line to the partner (except when a static picture was shown). Audio and video communication was implemented via low-latency streams (180 ms across laboratories). See further details of the equipment and software implementation later in this section and in Supplementary material.

#### 2.3.2 Audio and video

Audio was captured from both participants with stage microphones (HSE-150/SK) attached to IMG Stageline EMA-1 adapters and sampled by ESI Maya22 audio interfaces at 44100 Hz. Video was captured by webcams (Logitech C925e; 1080p resolution, 30 frames per second, 78 degrees visual angle) placed on the top of the screens.

#### 2.3.3 Eye tracking

Participants were equipped with a wearable eye tracker (Pupil Core, Pupil Labs GmbH, Berlin, Germany). This eye tracker has two cameras, the so-called “world camera” recording the scene seen by the participant, and the “eye camera” recording the diameter of the right pupil and the gaze direction of the right eye. The sampling rate was 60 Hz for the world camera, and 120 Hz for the eye camera (see further details in Supplementary material).

#### 2.3.4 Motion capture

For motion capture, an inertial motion capture system was used (Perception Neuron 32 System, Noitom Ltd., Beijing, China) with 11 sensors (each containing an accelerometer, a magnetometer, and a gyroscope) on the upper-body. The sampling rate was 120 Hz. In the current study, only data from the sensor on the head were analyzed, because (1) head movements are rich in communicative cues, (2) participants could only see each other from the shoulders up, and (3) participants were using a computer mouse to interact with the screen (which further limits the communicative movements, such as hand gesturing). The Axis Neuron software was used to read sensor data and to derive positions and kinetic measures of the body parts.

#### 2.3.5 Questionnaire on the quality of interaction and the perception of the partner

Participants’ subjective experience of the conversation partner and the quality of the interaction was assessed after each BG round using a short questionnaire (13 items) designed by the authors. The questionnaire contained statements about the other player (e.g., “I like my partner,” “I feel like my partner and I were in sync during the previous task,” “During the previous task, I often disagreed with my partner”) and about the task and the conversation in general (e.g., “The previous task was pleasant/enjoyable,” “I think that the previous conversation was smooth and natural”). A 7-point Likert scale was used for each questionnaire item. Exploratory factor analysis revealed no well-defined factors within the questionnaire, therefore, individual items were used in the statistical analyses (see English translations of all items in Table 2 with the original Hungarian items in Supplementary Table 2, where the subjective outcome variables are listed).

**Table 2:** Subjective communication outcome measures.

Name	Questionnaire items in English and indication of how the Likert scale values for the pair were aggregated
Perceived_Difficulty	I think that the previous task was hard. - MEAN
Naturalness_of_Conversation	I think that the previous conversation was smooth and natural. - MEAN
Rapport	My partner and I were on the same wavelength during the previous task. - MEAN
Liking	I like my partner. - MEAN
Disagreement	During the previous task, I often disagreed with my partner. - MEAN
Perceived_Synchrony	I feel like my partner and I were in sync during the previous task. - MEAN
Difference_in_Leading	I was leading the conversation during the previous task. - DIFFERENCE
Pleasantness_of_the_Task	The previous task was pleasant/enjoyable. - MEAN
Surprisal_of_Behavior	My partner’s behavior was surprising during the previous task. - MEAN
Truthfulness	I always told the truth during the previous task. - MEAN
Individual_Task_Performance	I think I had a bigger profit overall during the previous task. - DIFFERENCE
Efficiency_in_Trading	My partner and I exploited every opportunity for a trade. - MEAN
Predictability_of_the_Partner	My partner was playing the game in a predictable manner. - MEAN

MEAN and absolute DIFFERENCE were calculated from responses on the Likert scale.

### 2.4 Communication outcome measures

#### 2.4.1 Task performance in the BG/measures of communication gain and efficacy

Participants’ task in the BG was two-fold: they were instructed to increase their wealth as much as possible and to collect the must-have tokens in a given quantity. We defined communication gain as a wealth-related variable. Because the current BG was a non-zero-sum game, both participants could increase their wealth within the same round. Thus, the mean increase of wealth (relative to the initial mean wealth) across the two players, assesses how well the players could exploit the trading opportunities. In turn, communication efficacy is assessed by measuring how fast the pair achieved their goals. This was operationalized as the time spent to collect all the must-have tokens (averaged across the two players) and the total time it took for the participants to finish the game (i.e., when they both thought that further increase of wealth was not possible). For both communication gain and efficacy, we also assessed the asymmetry between the partners by calculating the absolute value of the difference between the wealth they gathered and the time they used to reach their set goals. Asymmetry in the latter was calculated only for game rounds where both players collected all must-have tokens (excluding rounds in which neither or only one player collected the must-have tokens). The ratio of these was 95.16% in the first round, 80.65% in the second, 87.40% in the third, and 59.52% in the fourth. All communication outcome measures were calculated separately for each round of the BG (see Table 3 for a summary of them).

**Table 3:** Objective communication outcome measures.

Type	Name	Definition
Communication gain	Mean_Wealth_Increase	Increase of wealth relative to the initial wealth (averaged across players).
	Difference_in_Wealth_Increase	Absolute difference between the players in increase of wealth relative to the initial wealth.
Communication efficacy	Mean_Goal_Time	Time spent to collect all must-have tokens (averaged across players).
	Difference_in_Goal_Time	Absolute difference between players in time spent to collect all must-have tokens.
	Total_Time	Time spent on BG.

#### 2.4.2 Subjective measures of communication outcome

Questionnaire items after each BG round (13 items) were used as subjective communication outcome variables (Table 2, for the original Hungarian versions of the items, see Supplementary Table 2).

Outcome measures were thus available separately for each pair of participants and BG round. Correlations between the communication outcome measures (both subjective and objective) are shown in Supplementary Figure 3.

### 2.5 Measures assessing interpersonal coordination (IC)

#### 2.5.1 Gaze coordination and pupil size coordination

Pupil diameter and gaze position within the picture recorded by the world camera was estimated using the automatic 3D pupil detection and gaze mapping algorithms implemented in the Pupil Capture (version 3.4.0) software (Kassner et al., 2014). Preprocessing of the raw pupil diameter data was necessary to correct for data loss due to blinks and other measurement artifacts. This preprocessing included confidence thresholding, diameter thresholding, filtering based on standard deviation, spike removal, removal of small clusters of data points, linear interpolation, and low-pass filtering. The effect of brightness on pupil diameter values was regressed out (see further details in the Supplementary material). Pupil size coordination between the two interlocutors was then estimated using dynamic time warping (DTW, Müller, 2007; applied to pupil size coordination by Wohltjen and Wheatley, 2021; although our implementation used Euclidean distance as a local distance measure instead of cosine similarity) implemented in Python, using the DTAIDistance package (version 2.3.10, Meert et al., 2022) with the default parameters. The window size was 6 s with a step size of 3 s. The DTW algorithm returns the overall cost of aligning (warping) two continuous signals that may be offset in time, thus indicating the dissimilarity (also referred to as the distance) of two signals for a given window. As a final step, we averaged the DTW distance values over time to obtain a single measure of overall pupil size coordination.

Gaze positions were considered only for valid fixations, i.e., gaze position data were discarded if the corresponding pupil diameter samples were removed during any of the steps mentioned above. For gaze tracking, 6 areas of interest (AOIs) were defined on the screen: the area for available tokens on the right side, the area for available tokens on the left side, the area for tokens marked to be sold, the area for tokens marked to be bought, the bargain button, and the face of the other participant. A gaze coordination episode was defined as the time interval within which both subjects were fixating on the same AOI. The gaze coordination ratio was calculated as the total duration of all gaze coordination episodes divided by the duration of the BG round (see further details in Supplementary material).

#### 2.5.2 Motion coordination

Motion coordination between the two interlocutors was analyzed based on head motion, only. Motion coordination was estimated by Pearson’s correlation of motion energy (calculated as the squared velocity integrated within a sliding window) of the two participants’ heads. A window size of 6 s and a step size of 8.33 ms (1 sample) was used. The window size was chosen considering the frequency range of head motion coordination (Fujiwara and Daibo, 2016) and the possible time lags between the interlocutors’ head motion (Tschacher et al., 2014; Ramseyer and Tschacher, 2011). Longer window sizes enable the capture of coordination at longer lags and lower frequencies, but smear high-frequency changes in the signals (see further details in Supplementary material).

#### 2.5.3 Characterizing prosody and the structure of the conversation

After minimal preprocessing of the audio streams (see further details in Supplementary material) an automated speech transcriber trained on Hungarian corpora (BEA Speech Transcriber - BEAST, Mihajlik et al., 2022) was employed for generating transcripts from audio recordings. Transcripts were then manually corrected for errors by trained university students and assistants working for the research group.

Precise speech on- and offset values were obtained using the Silero Voice Activity Decoder (Silero-VAD) model (Silero Team, 2024) in conjunction with the corrected transcripts. The final speech time series data were sampled at 200 Hz. Pitch and voice intensity values were then calculated for speech segments using Parselmouth (version 0.4.4) (Jadoul et al., 2018), a Python library for Praat (Boersma and Weenink, 2021).

#### 2.5.4 Prosodic coordination

When measuring alignment of speech prosody one has to consider that speakers do not accommodate to the other person immediately due to the reactive nature of conversations. Therefore, to estimate the level of prosodic alignment between participants, we calculated Pearson’s correlations from averages calculated within a moving window (also referred to as the TAMA [time-aligned moving average] method: Kousidis et al., 2008), separately for vocal pitch and intensity. The window length was 25 s and the step size 12.5 s. Thereby, almost all windows contained at least two full turns as the average speaker turn length was 3.24 s. This provided a good trade-off between the number of data points and the number of captured speaker turns. Pair-level differences were calculated to characterize asymmetries between the interlocutors (see the extracted variables in Table 4, “Prosodic coordination”).

**Table 4:** Predictor variables for modeling communication outcome.

Type	Name	Definition
Pupil size coordination	Pupil_Size_Coordination	DTW distance of pupil diameter time series (averaged over time).
Gaze coordination	Gaze_Coordination	Total duration of all gaze coordination episodes divided by the duration of the BG round.
Motion coordination	Motion_Coordination	Correlation between squared velocity values integrated in a moving window.
Prosodic coordination	Intensity_Correlation	Correlation between the speech intensity values of the interlocutors averaged within the moving window.
	Intensity_Difference	Absolute difference between the mean speech intensity of the interlocutors.
	Intensity_SD_Difference	Absolute difference between the standard deviation of speech intensity of the interlocutors.
	Pitch_Correlation	Correlation between the speech pitch values of the interlocutors averaged within the moving window.
	Pitch_Difference	Absolute difference between the mean speech pitch of the interlocutors.
	Pitch_SD_Difference	Absolute difference between the standard deviation of speech pitch of the interlocutors.
Conversation structure coordination	Speech_Rate_Correlation	Correlation between the speech rates (number of syllables divided by turn length) of the interlocutors, averaged within the moving window.
	Speech_Rate_Difference	Absolute difference between the overall speech rates (overall number of syllables divided by total time spent speaking) between the interlocutors.
	Speech_Amount_Correlation	Correlation between the number of syllables spoken by the interlocutors averaged within the moving window.
	Speaking_Time_Difference	Absolute difference between the relative speaking times (relative to the total time of the conversation) of the interlocutors.
	Backchannel_Rate_Difference	Absolute difference between the backchannel response rates (shorter than 1 s speech segments per minute) of the interlocutors.
	Interrupt_Rate_Difference	Absolute difference between the interruption rates (at least 0.5 s overlap of speech at turn ends, per minute) of the interlocutors.
	Pause_per_Minute_Difference	Absolute difference between the mean pause number per minute of the interlocutors. Pauses were defined as short, at least 0.2 s silences in a speaker’s turn.
	Pause_per_Speech_Difference	Absolute difference between the mean pause ratios within the speech-turns (compared to the length of speech) of the interlocutors.
	Pause_per_Turn_Difference	Absolute difference between the overall pause number per turn ratios of the interlocutors.
	Response_Time_Adaptation Difference	Absolute difference between the response time (length of silence before the speaker starts the turn) cross-correlations across subsequent turns of the interlocutors.
	Response_Time_Adaptation	Mean of the two interlocutors’ response time cross-correlations across subsequent turns.
	Response_Time_Difference	Absolute difference between mean response times of the interlocutors.
	Turn_Length_Adaptation _Difference	Absolute difference between turn length cross-correlations across subsequent turns of the interlocutors. Turn length was defined as the duration of the speaker’s uninterrupted speech (excluding backchannels and pauses).
	Turn_Length_Adaptation	Mean of the two interlocutors’ turn length cross-correlations across subsequent turns.
	Turn_Length_Difference	Absolute difference between the mean turn lengths of the interlocutors.
	Turn_Length_SD_Difference	Absolute difference between the standard deviation of turn length of the interlocutors.
	Turn_Rate_Difference	Absolute difference between the mean turn rate (number of turns per minute) of the interlocutors.
LSM	LSM	Overall LSM (language style matching) between interlocutors, averaged across the speech segments.
Manipulations	BG_Round	First, second, third, or fourth round of the bargaining game.
	Condition	Experimental condition.
	Time_before_BG_Round	Time in seconds spent in the game by the beginning of the current round.
Demographics	Age_Difference	Absolute age difference between the interlocutors.
	Mean_Age	Mean age of the interlocutors.
	Gender	Gender of the interlocutors (either male or female, as only same-gender pairs were studied).
	Handedness	Handedness of the interlocutors (left, right, or mixed).

#### 2.5.5 Coordination in the structure of the conversation

Structural characteristics of speech-turns within a conversation (such as turn length, speech rate, etc.), may also become coordinated between interlocutors over the course of a conversation, and these variables have been shown to influence the outcome of the conversation (Di Stasi et al., 2024). We extracted several features of the structure of conversations from the speech signal: turn length, speaking times, speech rate, pauses, backchannels, interruptions and response times. We investigated the coordination of these features by calculating (1) the overall difference between the interlocutors in these features - describing the similarity between the interlocutors, and (2) the correlation between these features across the interlocutors - describing a turn-by-turn adaptation. For the latter we calculated the cross-correlations between subsequent turns (1 lag) and calculated pair-level means and differences for the correlation coefficients. The mean correlation value is an indicator of how much the pair coordinated their structural conversation characteristics, while the difference variables describe asymmetry in adaptation between the interlocutors (e.g., if one participant adapted more to the other than vice versa). For speech rate and speech amount, we calculated correlation identically to prosodic variables.

#### 2.5.6 Language style matching

Language Style Matching (LSM) is a technique that assesses the stylistic and syntactic similarities in language use across people by measuring the use of function words (Ireland and Pennebaker, 2010). The function word categories usually assessed are personal pronouns, impersonal pronouns, conjunctions, adverbs, articles, auxiliary words, negations and quantifiers. LSM is calculated for each function word category using the following formula, where X denotes the function word category under investigation and X_1_ and X_2_ are the number of function words used by the two speakers:

$$\mathrm{LS}M_{X}=1-[(|X_{1}-X_{2}|)/(X_{1}+X_{2}+0.0001)]$$

This yields a value between 0 and 1, where the closer the number is to 1, the higher the similarity between speakers. Then, to obtain a composite value of LSM, the results of the eighth categories are averaged.

In order to calculate LSM between participants, speech transcriptions were segmented to identical 2-min long speech segments for both participants. We used HuSpaCy (Orosz et al., 2023), a Hungarian Natural Language Processing (NLP) library using SpaCy models to assess the grammatical categories of words and to calculate their occurrence in the texts. Then, we calculated LSM between speakers for each corresponding segment of speech and averaged them to estimate the overall LSM of a pair.

Variables included in the statistical models set up to predict communication success (see next section) are summarized in Table 4. Each variable was established separately for each pair of participants, separately for each round of the BG. Correlations between these predictor variables are shown in Supplementary Figure 4.

### 2.6 Statistical analysis

#### 2.6.1 Data preparation

All variables included in the statistical analysis were manually screened for outliers. Variables measuring the absolute difference between interlocutors as well as time- and velocity-related variables were log-transformed to address skewness in the data. Variables estimated by Pearson’s correlation were converted to approximately normal distribution by Fisher’s Z-transformation. Observations (a given pair in a BG round) with missing data in any of the predictor variables were omitted from the analyses needing the missing data (the numbers of observations with missing predictor variable data are given in Supplementary Table 3).

#### 2.6.2 Validation of coordination measures

The first step of the statistical analysis was the comparison of real and pseudo pairs for each tested measure of coordination (see Table 4, except for the Manipulations and the Demographics sections of the table). Pseudo pairs are random pairings of participants, who did not participate in the same experimental session and thus did not play the BG together. That is, they played the same game in the same context, but with a different partner. Thus, if some measure significantly differs between real pairs and pseudo pairs, it reflects coordination of the given measure due to the actual interaction during the experimental session, as opposed to general contextual commonalities. Pseudo pairs were restricted to being selected from the same condition (Baseline, Unfamiliar, Unimodal) and BG round (1, 2, 3, 4). Therefore, a recording of a given participant in a given round of the game was paired only with recordings of individuals playing in the same condition and the same round of the game. Real pairs and pseudo pairs were compared with one-tailed random permutation *t*-tests (Janssen, 1997), with an alpha-level of 0.05 and 10^5^ permutations (with Mkinfer 1.2 in R; Kohl, 2024). Holm’s method was applied to the significance values in order to control the family-wise error rate (FWER; Holm, 1979). Only measures surviving the validation, i.e., showing significantly different values for real pairs compared to pseudo pairs, were included in the statistical model below (see *3.1 Interpersonal coordination: real vs. pseudo pair comparisons* for the surviving measures).

#### 2.6.3 Testing the effects of experimental conditions on IC measures

To explore the effect of the experimental conditions we employed Random Forest classification (Breiman, 2001) using the caret package in R (version 7.0–1; Kuhn, 2008). Random Forests are ensemble methods that build multiple decision trees and aggregate their predictions, offering high classification accuracy and the ability to estimate the importance of each predictor variable (Breiman, 2001). We used this method to assess how well experimental conditions can be differentiated using IC measures. Because both the Unfamiliar and the Unimodal condition differed from the Baseline condition in a single (but different) feature, classification was tested between pairs of conditions: the Unfamiliar vs. the Baseline condition and the Unimodal vs. the Baseline condition. The models included all IC measures (Table 4, except for Manipulations and Demographics). The models were trained using ten-fold cross-validation, with 500 trees to ensure model robustness and reduce overfitting. The “mtry” parameter, which determines the number of predictors randomly sampled at each split in the forest, was tuned automatically, with the optimal value selected based on the models’ cross-validated classification accuracy. Variable Importance analysis was also carried out using the same version of the caret package in R. Variable Importance analysis in Random Forest models is useful to assess the contribution of each predictor to the model’s performance: it ranks variables based on their influence on reducing classification error across the trees in the forest.

#### 2.6.4 Testing the effects of difficulty on IC measures

A similar Random Forest classification approach was used to test the effects of the difficulty manipulation on measures of IC. We investigated whether and which IC measures can be used to reliably differentiate between the BG rounds. For this analysis, we used a single classification model with all four levels entered (BG round 1 to 4). The models again included all IC measures (Table 4, except for Manipulations and Demographics); cross-validation and parameter settings of the model as well as the Variable Importance analysis were identical to the testing of the effects of the experimental conditions (Section 2.6.3).

#### 2.6.5 Testing the relationships between coordination and outcome variables

We tested the effects of the manipulation, demographic, and the surviving coordination variables (see previous section) on the communication outcome variables (Tables 2, 3) together in a multivariate approach. Because the interdependence pattern across our relatively large number of predictors might lead to poor estimates, a two-step analysis was carried out on the data.

First, the number of potential predictors was restricted by fitting linear models with LASSO (Least Absolute Shrinkage and Selection Operator) regularization separately to each communication outcome measure (with glmnet 4.1.8 in R; Friedman et al., 2010; Tay et al., 2023). The LASSO method imposes a penalty on regressors, forcing small coefficients to equal zero, yielding a robust model fit even in the presence of collinearity (Helwig, 2017). The *λ*-parameter (penalty strength parameter) was estimated by five-fold cross-validation, selecting the value that gives the most regularized model such that the cross-validated error is within one standard error of the minimum (1-standard-error λ). As the cross-validation might yield different results depending on the initial random splitting of the data into the training and validation set, the five-fold cross-validation was run 11 times for each outcome variable and the median of the resulting 11 λ values was used.

Second, a linear-mixed model (LMM, with lme4 1.1–35 in R, Bates et al., 2015) including the final predictors from the LASSO models in the first step have been refitted to the full data set with random intercepts added for pairs of interlocutors. This step was included to improve the interpretability of the final results and to account for random effects of the pairs of interlocutors (see, e.g., Di Stasi et al., 2024 for a similar approach). Statistically significant (alpha-level 0.05) predictors are reported with the effect sizes estimated by f^2^ (Lorah, 2018).

## 3 Results

### 3.1 Interpersonal coordination: real vs. Pseudo pair comparisons

While not all coordination variables survived the validation step, we found significantly different IC for real pairs compared to pseudo pairs in all of the categories of the coordination variables (Table 5). Altogether, 12 of the 27 IC variables emerged as significantly different from pseudo pairs (for the IC measures that did *not* survive the validation step see Supplementary Table 4). As some variables were transformed before the statistical analysis (log- or Z-transformed), for these variables, Table 5 shows both the original mean and standard deviation (upper) and the transformed mean and standard deviation (lower).

**Table 5:** Results of the permutation tests comparing real and pseudo pairs for IC variables.

Variable name	Effect size (Cohen’s *d*)	Mean (SD) real	Mean (SD) pseudo	Direction of the effect	Significance
Pupil_Size_Coordination	−0.188	12.628 (2.196)	13.063 (2.314)	*P >* R	**
Gaze_Coordination	0.598	8.865 (4.856)	6.819 (3.340)	*P <* R	***
Head_Motion_Coordination	0.564	0.083 (0.173) 0.088 (0.186)	0.009 (0.128) 0.009 (0.136)	*P <* R	***
Intensity_Correlation	0.583	0.199 (0.314) 0.226 (0.365)	0.006 (0.331) 0.007 (0.377)	*P <* R	***
Intensity_SD_Difference	−0.21	0.664 (0.685) −0.550 (0.759)	0.808 (0.765) −0.385 (0.787)	*P >* R	***
Pitch_Correlation	0.314	0.115 (0.298) 0.128 (0.338)	0.011 (0.327) 0.012 (0.371)	*P <* R	***
Speech_Rate_Correlation	0.236	0.116 (0.303) 0.129 (0.343)	0.037 (0.325) 0.041 (0.370)	*P <* R	***
Speech_Amount_Correlation	−0.484	−0.157 (0.361) −0.181 (0.444)	0.002 (0.330) 0.002 (0.375)	*P >* R	***
Interrupt_Rate_Difference	−0.52	0.249 (0.246) -1.271 (0.665)	0.407 (0.367) −0.913 (0.690)	*P >* R	***
Response_Time_Difference	−0.623	0.352 (0.314) -1.012 (0.663)	0.649 (0.545) −0.548 (0.749)	*P >* R	***
Turn_Rate_Difference	−0.949	0.621 (0.513) −0.598 (0.785)	1.583 (1.171) 0.219 (0.864)	*P >* R	***
LSM	0.582	0.624 (0.074)	0.573 (0.087)	*P <* R	***

Only the variables with significant differences between real and pseudo pairs are shown here. For variables, where a transformation was applied before statistical analysis, the upper value shows the original mean and standard deviation (SD), the lower value shows the transformed mean and SD. The direction of the effect is shown as R[eal] > P[seudo] or *P >* R; see main text where explanation is needed to interpret the statistical finding. *p < 0.05, **p < 0.01, ***p < 0.001.

Pupil_Size_Coordination was significantly higher for real pairs compared to pseudo pairs - note, that Pupil_Size_Coordination was calculated as DTW distance, which means that the smaller the distance between two signals, the larger the coordination, hence the negative effect size. Significantly more negative correlation was found for speech amount in real than pseudo pairs, which most likely results from the turn-taking structure, present in real but absent in pseudo pairs.

### 3.2 Predicting objective measures of communication outcome

Out of the total 27 IC variables, the above mentioned 12, which showed significant differences among real and pseudo pairs (Table 5) were entered into the LASSO selection and subsequent LMM models of the objective communication outcome variables (Table 3), along with the demographic and manipulation-related variables (Table 4).

Out of the five variables objectively assessing communication outcome, Mean_Wealth_Increase and Difference_in_Wealth_Increase characterized communication gain, while Mean_Goal_Time, Difference_in_Goal_Time, and Total_Time characterized communication efficacy. From the communication gain measures, no significant predictors were obtained for the Difference_in_Wealth_Increase, while only BG_Round emerged as a significant predictor of the Mean_Wealth_Increase variable, showing that with increasing difficulty, the amount of wealth gained decreased. As no IC measure emerged as a significant predictor for the communication gain measures, the related models were not analyzed further.

**Figure 3:**
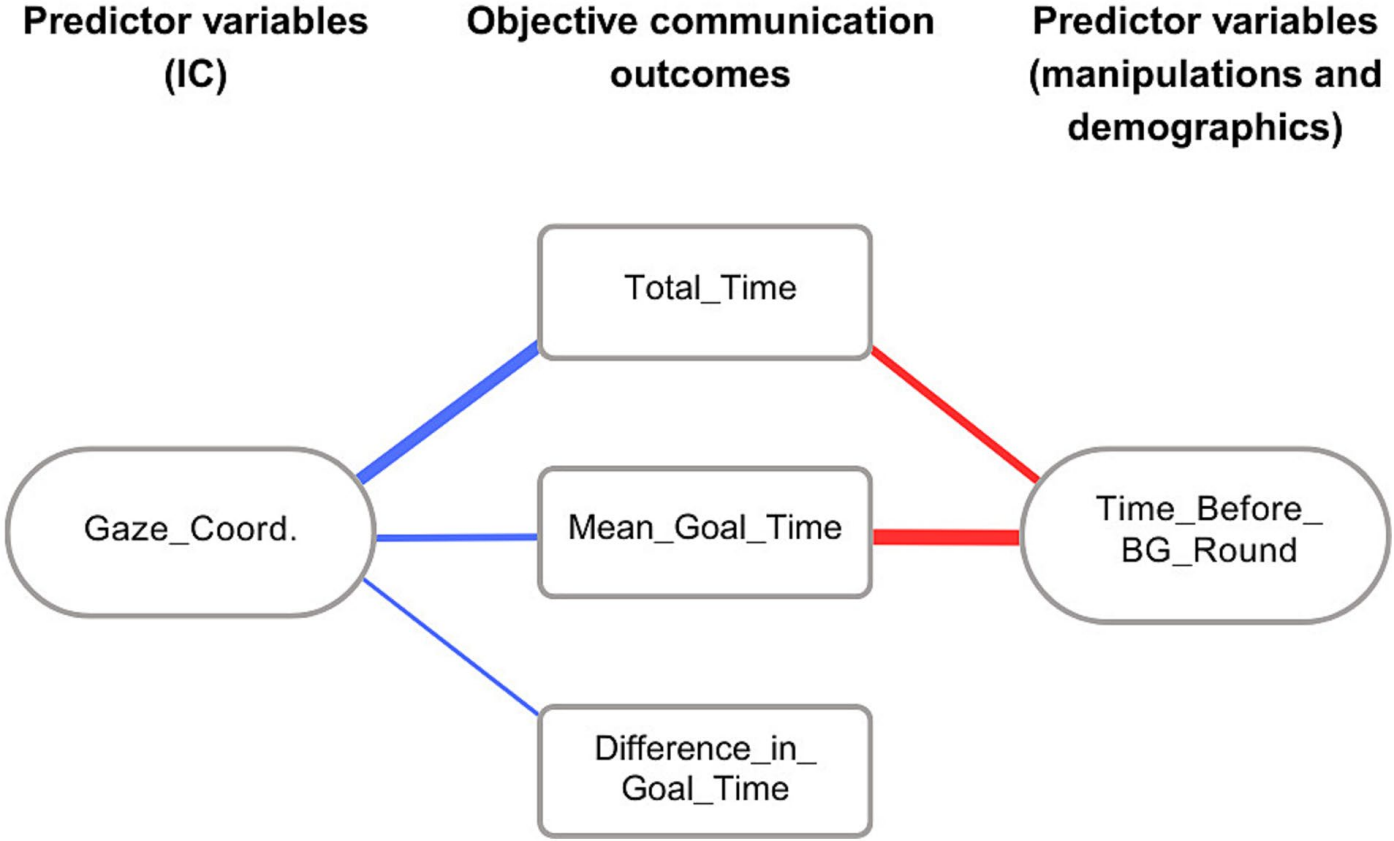
Graphical illustration of the relationship between objective measures of communication efficacy and the significant predictor variables. Rectangular nodes: measures of communication efficacy. Rounded nodes: significant predictor variables. Edge color denotes the direction of the relationship between the variables (red: positive, blue: negative), edge thickness denotes the effect size (larger effect size corresponds to larger thickness).

For the communication efficacy measures, Gaze_Coordination emerged as a significant predictor for: Mean_Goal_Time (*p <* 0.01, f^2^ = 0.038), Difference_in_Goal_Time (*p <* 0.05, f^2^ = 0.018) and Total_Time (*p <* 0.001, f^2^ = 0.087). The Time_Before_BG_Round variable was a significant predictor of Mean_Goal_Time (*p <* 0.001, f^2^ = 0.114) and Total_Time (*p <* 0.001, f^2^ = 0.055). The relationship (direction and effect size) between objective measures of communication efficacy and significant predictor variables are graphically illustrated as a network in Figure 3. The figure shows that better Gaze_Coordination resulted in more efficient task performance (shorter time spent to reach goals) and better collaboration (smaller difference between the partners in reaching their goals).

The effects of the time at which the round started may mix together two different causes: the difficulty of the round (as later rounds were more difficult) and the speed at which the pair played the game (slower pairs reached later rounds at a later time). As both of these sources can be assumed to have negative effects on the efficacy of communication (positive effects on the temporal measures), we carried out a *post-hoc* analysis of individual BG rounds. The analysis showed that the variable Time_before_BG_Round had a significant effect on communication efficacy even when the factor of increasing difficulty was eliminated: a significant positive effect on Total_Time for the second BG round (*p <* 0.001, f^2^ = 0.055), and a significant positive effect on Total_Time (*p <* 0.001, f^2^ = 0.05) and on Mean_Goal_Time (*p <* 0.001, f^2^ = 0.114) for the third BG round (for the first round, the Time_before_BG_Round is always zero, for the fourth round no significant effect was found due to the relatively small number of pairs completing the fourth round). Ultimately, pairs had a propensity to spend more time on a BG round if previous games were also longer, which suggests that pairs play the game at their own pace (i.e., pairs who were slower in previous games remain slower in the current game).

LMM results including the model fit for objective measures of communication efficacy and significant predictor variables are summarized in Supplementary Table 5.

### 3.3 Predicting subjective measures of communication outcome

**Figure 4:**
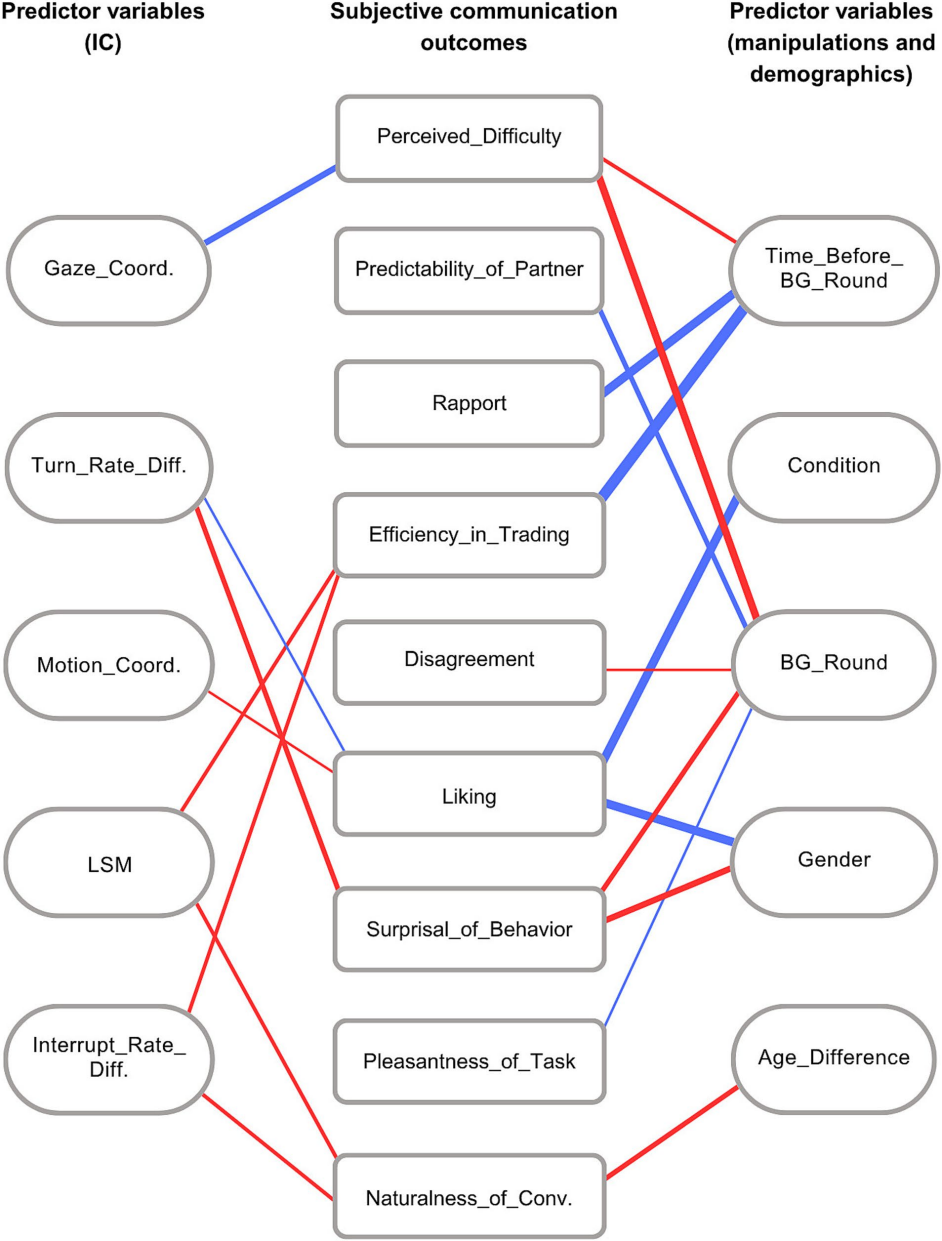
Graphical illustration of the relationship between subjective communication outcomes and the significant predictor variables. Rectangular nodes: subjective communication outcomes. Rounded nodes: significant predictor variables. Edge color denotes the direction of the relationship between the variables (red: positive, blue: negative), edge thickness denotes the effect size (larger effect size corresponds to larger thickness).

IC (surviving the validation phase), demographic, and manipulation variables were included in the models aimed to explain the subjective outcome variables. Significant relationships are illustrated as a network in Figure 4. Here we first describe the effects of the IC variables, then that of the other variables.

LMM results including the model fit for subjective communication outcomes and significant predictor variables are summarized in Supplementary Table 6. Note that for the models containing both BG_Round and Time_Before_BG_Round, two effect sizes are shown for these variables. These variables explain somewhat, but not fully overlapping parts of the total variance (cf. the *post-hoc* analyses at the end of section *3.2 Predicting objective measures of communication outcome*). As a result, leaving only one of them out of the model (as is normally done for the calculation of f^2^) only slightly changes the model fit. The first effect size in Supplementary Table 6 reflects the standard calculation of f^2^, whereas the second effect size was calculated based on a model excluding BG_Round for Time_Before_BG_Round and excluding Time_Before_BG_Round for BG_Round.

#### 3.3.1 IC predictors of subjective communication outcomes

We found that higher Gaze_Coordination was linked with reduced Perceived_Difficulty (*p <* 0.001, f^2^ = 0.037), while better Motion_Coordination was linked with higher score in Liking (*p <* 0.05, f^2^ = 0.005). Out of the 8 variables describing coordination in the structure of conversations, only two predicted subjective evaluations significantly. We found that Turn_Rate_Difference was positively associated with Liking (*p <* 0.05, f^2^ = 0.007) and negatively with Surprisal_of_Behavior (*p <* 0.01, f^2^ = 0.022). Somewhat surprisingly, we observed that Interrupt_Rate_Difference was positively associated with Naturalness_of_Conversation (*p <* 0.05, f^2^ = 0.012) and Efficiency_in_Trading (*p <* 0.05, f^2^ = 0.013). That is, the higher the difference was between the interruption rates of the two interlocutors, the more likely they rated the conversation as natural and effective. Finally, we also found that LSM (our measure of alignment in language style) had a significant positive effect on Naturalness_of_Conversation (*p <* 0.01, f^2^ = 0.019) and Efficiency_in_Trading (*p <* 0.05, f^2^ = 0.014).

#### 3.3.2 Other predictors of subjective communication outcomes

For the variables describing the experimental manipulations, we found an effect of Condition on Liking (*p <* 0.001, f^2^ = 0.086). Participants reported the highest scores for how much they like their counterpart in the Baseline condition (mean = 6.507), followed by the Unimodal condition (mean = 6.292), with the lowest reports for Liking in the Unfamiliar condition (mean = 6.016). This result indicates that pairs who did not have the introductory conversation before the BG liked their partner less, compared to pairs who did have a chance to meet before the BG started. In order to specify this effect, we asked whether it was more pronounced in the earlier rounds of the BG compared to later games by running a two-way ANOVA with BG_Round as within-subject factor and Condition as between-subject factor. We found no significant interaction (*F*(6, 240) = 0.286, *p >* 0.5, partial eta-squared = 0.007), which suggests that the initial difference caused by the familiarity manipulation did not significantly change during the time spent in the BG. As expected, BG_Round and Time_before_BG_Round emerged as predictors for several outcome measures. In particular, increasing difficulty in the BG (BG_Round) resulted in higher scores on Perceived_Difficulty (*p <* 0.001, f^2^ = 0.051), Disagreement (*p <* 0.001, f^2^ = 0.003), and Surprisal_of_Behavior (*p <* 0.001, f^2^ = 0.028), whereas lower scores on Pleasantness_of_the_Task (*p <* 0.05, f^2^ = 0.003) and Predictability_of_the_Partner (*p <* 0.001, f^2^ = 0.020). We also found that with increasing time spent in the BG (Time_before_BG_Round), Perceived_Difficulty was higher (*p <* 0.05, f^2^ = 0.017), while Rapport (*p <* 0.001, f^2^ = 0.070) and Efficiency_in_Trading (*p <* 0.001, f^2^ = 0.156) were lower.

For the demographic variables, we found that Gender significantly predicted Liking (*p <* 0.001, f^2^ = 0.096): male participants gave lower scores for how much they liked their counterpart during the BG rounds. Male interlocutors also responded with higher scores for Surprisal_of_Behavior (*p <* 0.05, f^2^ = 0.038). Additionally, we found that Age_Difference had a significant positive association with Naturalness_of_Conversation (*p <* 0.05, f^2^ = 0.027), however it did not emerge as a predictor for any of the other outcome measures. While we acknowledge that females and males might have had slightly different subjective impressions of communication quality and their partner, or expressed their impressions differently, it should be noted that our study was not suitable to properly investigate gender effects, as the number of female and male participants was not balanced. Further, the age of most of our participants fell into a narrow range (93% of our participants were between 18 and 25 years) and the effect of Age_Difference did not change after excluding pairs with a participant over 30 years of age. Therefore, the effects of these demographic variables will not be discussed further in Section *4 Discussion*.

### 3.4 Assessing the effects of the experimental conditions on IC

**Figure 5:**
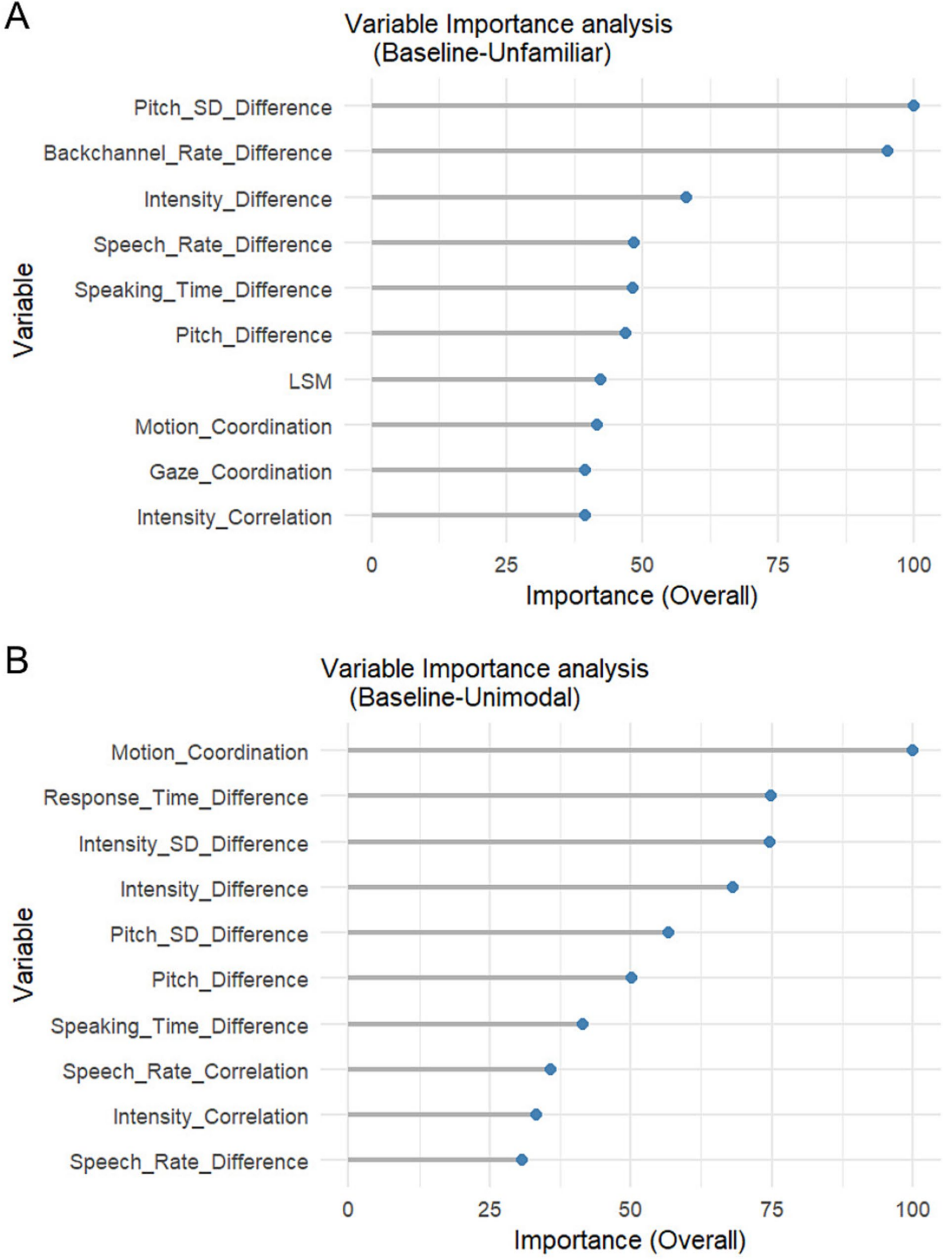
Variable importance analysis from the random forest models on the familiarity and modality manipulations. Separate models were used for the comparison between the baseline and the unfamiliar condition **(A)**, and between the baseline and the unimodal condition **(B)**. Overall importance is depicted on a relative scale from 0 to 100, where 100 is assigned to the most important variable, followed by other variables in proportion to the top variable. The first 10 (out of 27) variables are shown.

For the comparison between the Baseline and the Unfamiliar condition, the best performing Random Forest model reached an accuracy of 66.25% (using mtry = 2, selected via cross-validation), with a Kappa statistic of 0.31, indicating a moderate effect. The most important variable selected for classification was Pitch_SD_Difference (Unfamiliar M = 1.89, SD = 0.914; Baseline M = 2.21, SD = 0.909), followed by Backchannel_Rate_Difference (Unfamiliar M = −1.68, SD = 0.517; Baseline M = −1.55, SD = 0.529) and Intensity_Difference (Unfamiliar M = 1.01, SD = 0.997; Baseline M = 1.14, SD = 0.987) (Figure 5A). Note that the M and SD values reflect the log-transformed variables.

For the comparison between the Baseline and the Unimodal condition, the best performing Random Forest model achieved an accuracy of 67.89% (with mtry = 2, selected via cross-validation), with a Kappa statistic of 0.36. Based on the Variable Importance analysis (Figure 5B), the most influential variables were Motion_Coordination (Unimodal M = 0.030, SD = 0.131; Baseline M = 0.106, SD = 0.206), followed by Response_Time_Difference (Unimodal M = −0.992, SD = 0.606; Baseline M = −0.944, SD = 0.718), and Intensity_SD_Difference (Unimodal M = −0.587, SD = 0.724; Baseline M = −0.517, SD = 0.775). Note that the M and SD values reflect the log-transformed variables.

### 3.5 Assessing the effects of task difficulty on IC

The best performing model reached an accuracy of only 27.27% (with mtry = 2, selected via cross-validation), with a Kappa statistic of 0.004. This indicates the lack of reliable effects of difficulty on IC in the current study. In light of this, the Variable Importance analysis will not be discussed further (but see the Supplementary Figure 5 for the results).

## 4 Discussion

Previous research has established that interpersonal coordination (IC) is a widespread phenomenon in social interaction. People coordinate across a variety of behaviors (eye gaze, movement, speech, etc.) and physiology (e.g., pupil size, heart rate, skin conductance or neural states), but the possible function(s) IC might serve in social interactions is poorly understood. The aim of the present study was to (1) investigate the ubiquity of IC (with multiple behavioral and physiological measures) in an unconstrained task-oriented verbal interaction between two partners, and (2) to assess the effects of IC on both objective and subjective outcomes of the conversation.

Regarding the presence of IC in on-line face-to-face communication, we showed that interlocutors coordinated their behavior on multiple levels - head motion, pupil size, gaze direction, some prosodic features, such as pitch and intensity, and several structural features of the conversation (speech rate, speech amount, interrupt rate, response times, turn rate and language style). Our first hypothesis was thus supported by the current data: we found significantly higher IC for real compared to pseudo pairs in all of the measured categories of behavior (cf. section *3.1 Interpersonal coordination: real vs. pseudo pair comparisons*, Table 5). The presence of IC in various forms is compatible with both of the two main previously proposed models of communication: IAT (Interactive Alignment Theory; Pickering and Garrod, 2004) and DST (Dynamical Systems Theory; e.g., Shockley et al., 2009, Friston and Frith, 2015).

In contrast, our second hypothesis which suggested that stronger IC predicts better communication outcomes, was only partially supported by the data. For the objective outcomes of the bargaining game, we found that while communication efficacy was predicted by Gaze_Coordination (but no other IC variable), no current IC variable predicted our measures of communication gain (indexed by the collected wealth). On the other hand, we found that some of the IC variables were associated with more positive subjective communication outcomes: Motion_Coordination and Turn_Rate_Difference predicted Liking, LSM predicted Efficiency_in_Trading and Naturalness_of_Conversation, while Gaze_Coordination emerged as a significant negative predictor for Percieved_Difficulty. However, we also found evidence for effects in the opposite direction; dyads with higher Interrupt_Rate_Difference (that is, lower coordination of interruptions) gave higher ratings for Naturalness_of_Conversation and Efficiency_in_Trading (see section *4.2.3 Coordination in the structure of conversation*). One should, however, note that also these variables showed higher coordination for real over pseudo-pairs. Thus, the direction of their effect on subjective communication outcomes rides on top of the significant coordination effect. Further, gaze coordination might have arisen between the partners as a natural consequence of how the BG task was designed (for a detailed discussion, see Section 4.2.1), thus its association with the outcome variables may not reflect a general relationship.

In Pickering and Garrod’s (2004) influential theory of alignment (IAT) the coordination at multiple levels of behavior is central to achieving mutual understanding and successful communication. From this point of view, the present results do not provide strong support for such mechanistic effects of IC. While our operationalization of communication success may not have been sufficiently effective (see, section *4.2 Associations between IC variables and the outcome measures of the interaction*), one would still expect at least some of the speech-related IC variables to have a positive relationship with the objective outcome measures. On the other hand, while the opposite-direction effects found for the subjective outcomes may appear to contradict a strict mechanical interpretation of IAT, they may be interpreted as stronger coordination between interlocutors assuming that they play different roles in the interaction (cf. section *4.2.3. Coordination in the structure of conversation*). Recent work emphasized that more complex tasks might require partners to use IC in a way that alternates between convergence and divergence (daSilva and Wood, 2024). That is, dyads might occasionally benefit more from dissimilar, unique behaviors that foster creative problem-solving. The current results suggest that while the effect of IC on performing a joint task may not be strong and obligatory (i.e., it may depend on the specifics of the task), IC (especially in speech-related features) may be more strongly associated with how interlocutors experience the interaction and their partner. Indeed, recent integrative theories (daSilva and Wood, 2024; Gordon et al., 2024) about the functions of IC proposed that distinct dimensions of aligned behavior/physiology serve different functions (e.g., accomplishing joint tasks vs. increasing social connection with the partner). On the other hand, the presence of IC for all measured categories of variables, including those more closely reflecting physiological functions (such as pupil size), suggests that IC may be an inherent emerging property of any communicative interaction.

Our final test assessed whether levels of IC distinguish between the levels of familiarity between the partners, the levels of task difficulty, and whether real-time visual information was available or not. While models based on IC variables distinguished between the current levels of familiarity and the presence vs. absence of real-time visual information with moderate accuracy, similar models performed poorly in distinguishing the different levels of task difficulty in the BG. Thus, familiarity between the partners and the availability of rich information of the partner’s behavior at least moderately modulate certain forms of IC, although neither did significantly affect communication success. These effects are compatible with the theoretical position of IAT with the qualification that not all effects on IC influence communication success at the same time. The effects of familiarity are also compatible with the DST approach.

Overall, the picture emerging from the current results is that some interpersonal coordination is likely an emergent property of on-line face-to face interactions (support for Dynamical Systems Theories). However, while some of the results are also compatible with the Interactive Alignment Theory, communication in the bargaining game did not provide strong evidence for a straightforward role of interpersonal coordination in face-to-face communication. On the other hand, the negative association between some of the current IC measures and some subjective outcome measures would also pose problems for theories based purely on DST. Rather, these could be accommodated by extending the Interactive Alignment Theory to acknowledge potentially different roles of the partners in a joint task situation (Fusaroli and Tylén, 2016; daSilva and Wood, 2024; Gordon et al., 2024; see also section *4.2.3 Coordination in the structure of conversation*).

In the rest of Section 4, we discuss in more detail the different types of IC-related effects found in the current study.

### 4.1 Interpersonal coordination

We observed coordination in pupil size between the interlocutors, a physiological measure modulated both by cognitive and emotional effects, even after removing the effects of brightness levels from the pupil size signal. Pupil size coordination did not correlate with gaze coordination (r = −0.07, *p >* 0.05), nor head motion coordination (r = −0.05, *p >* 0.05) suggesting that it did not reflect the gazing constraints of the task (i.e., looking at similar visual areas in the two labs). Thus, pupil size coordination might reflect shared attentional mechanisms across the interacting agents (Kang and Wheatley, 2017), even though our study does not provide direct evidence that attending to certain stimuli at the same time causes pupil diameters to covary.

Behavioral coordination was observed for participants’ gaze patterns, which suggests that through verbal communication players could direct each other’s attention to relevant areas of the screen. Head motion was another aspect of behavior that became coordinated during communicative exchanges, which is in line with studies emphasizing the ubiquity of temporal coordination of body movements under interactive contexts (Cornejo et al., 2017). Similarly, we found alignment in some prosodic features (pitch and intensity) and certain structural conversation dynamics (e.g., speech rate, interrupt rate, response times, language style, etc., but not for back-channel responses or the length of speech turns) between interacting individuals.

Thus, our data shows that IC, in general, is a robust feature of on-line face-to-face communication. The types of coordination observed in our experiment indeed stemmed from the interactions between the interlocutors, because testing them against a randomized control sample taken from the same experimental situation (see *2.6 Statistical analysis* for details) ruled out the possibility that IC has been imposed by the shared context, i.e., not requiring actual interaction between the partners. Overall, the presence of IC in various types of behaviors is compatible with both types of previously proposed models, IAT and DST.

### 4.2 Associations between IC variables and the outcome measures of the interaction

#### 4.2.1 Gaze coordination

Out of the tested IC measures, Gaze_Coordination was the only reliable predictor of communication efficacy in the BG. Pairs who showed higher coordination of gaze patterns were faster at solving the BG and needed less time to collect the must-have tokens. With higher gaze coordination, partners also collected the must-have-tokens at times closer to each other than pairs with lower gaze coordination (the Difference_in_Goal_Time was smaller). Thus, gaze coordination appears to index how well participants cooperated with each other. Gaze coordination was associated with not only the efficacy of communication, but also with how participants perceived the difficulty of the game (more gaze coordination leading to perceiving the game as easier). It is possible that the Gaze_Coordination variable mediated the effect of how long the game lasted on the partners’ perception of the difficulty of the game, which was supported by a *post-hoc* analysis (see Supplementary material). Previous studies found that alignment in behaviors tightly linked to the task are associated with performance in a joint task (see Jermann and Nüssli, 2012 vs. Coco et al., 2018). The current BG required partners to negotiate bargains for various tokens in their inventory, prompting them to look at their own token prices at the same time. Thus, the association between gaze coordination and the efficacy of completing the task may be a consequence of the BG task. Note, however, that even if gaze coordination is not a task-independent effect, it still reflects coordination between the partners.

#### 4.2.2 Head motion coordination

Head_Motion_Coordination showed a significant positive association with Liking (i.e., how much partners liked each other). This result is in line with several previous findings demonstrating the connection between motion coordination and prosocial effects. For example, finger tapping in synchrony with someone as opposed to tapping in asynchrony increases affiliation (Hove and Risen, 2009), motion coordination between therapist and patient increases perceived relationship quality and rapport (Ramseyer and Tschacher, 2011), while rhythmic similarity increases bonding in strangers (Fujiwara et al., 2020). As previously mentioned, IC of head movements is often discussed under a DST approach (e.g., Paxton and Dale, 2017). While DST does not propose IC to affect the outcome of the interaction, head-motion could reflect communicative actions such as nodding (and thus can also be regarded as a measure of IAT) or underlie other strictly communication-related IC phenomena (e.g., laughing).

#### 4.2.3 Coordination in the structure of conversation

Turn_Rate_Difference showed a significant negative association with Liking and a positive one on Surprisal_of_Behavior (i.e., to what extent the partner’s behavior felt surprising). Whereas the former may reflect that differences in speaking style (shorter vs. longer turns) could somewhat reduce sympathy between the interlocutors, the latter suggests that interlocutors with more similar speaking styles perceived the behavior of their partner more predictable. Thus these effects are compatible with those predicted by IAT with the assumption that more similar speaking styles may reflect better understanding between the interlocutors.

We also found that LSM had a significant positive association with the Naturalness_of_Conversation and the Efficiency_in_Trading items of the questionnaire. This finding is consistent with previous ones emphasizing the positive consequences of LSM in relation to cooperation as our Efficiency_in_Trading measure can be interpreted as a form of cooperation (Richardson et al., 2019; Van Swol and Carlson, 2017; Bayram and Ta, 2018), and to the perception of the conversation (Vergani, 2022; Liu et al., 2019). Thus, similarity of linguistic style may boost interlocutors’ subjective experience about the conversation flow and efficiency of collaboration. The latter is also interesting in light of the fact that LSM did not improve actual performance.

However, we also found that Interrupt_Rate_Difference showed significant positive association with Naturalness_of_Conversation and Efficiency_in_Trading, thus showing that larger differences between the partners led to more positive subjective outcomes, contradicting the general notion of IAT (i.e., that stronger IC leads to better outcome). Expectation for an association of an opposite direction was supported by previous research on the function of interruptions in conversation which showed that interlocutors who interrupt their partner often are less liked, but perceived as having higher status (Farley, 2008; Hall et al., 2005). Note, however, that, overall, partners aligned with each other in how often they interrupted their partner, as they showed more similar interrupt rates than pseudo pairs (Table 5). These results may rather fit with those of an increasing number of recent studies showing that more alignment does not necessarily lead to better outcomes. For example, Fusaroli and Tylén (2016) have shown that collective performance in a perceptual task could be better predicted by an ‘interpersonal synergy’ approach, rather than an ‘interpersonal alignment’ approach, with the former method taking into account the complementary structure of conversation, rather than just the overall similarity between partners (see also Amon et al., 2019 for a similar approach with triads and Paxton et al., 2014 on the positive effects of weaker IC of body movements in a joint tower-building task). Our participants also performed a joint task, which may suggest that some asymmetry in communication may be more optimal in a task setting, where complementary roles could be more beneficial than in less goal-oriented situations.

A possible qualification of this effect suggests that perhaps, the pair-average scores for the Naturalness_of_Conversation variable were driven by higher scoring from the partner, who interrupted more often than the other. To test this hypothesis, we conducted a *post-hoc* analysis (Pearson’s chi-squared test, see Supplementary Table 7)between individual interrupt rate of a participant and their individual score for the Naturalness_of_Conversation. However, the results showed that the null hypothesis of no relation could not be rejected (*p >* 0.1).

We also note that Interrupt_Rate_Difference, Turn_Rate_Difference, and LSM were not found to be significant predictors of any of the objective outcome variables and showed very low correlation with gaze coordination (−0.09 < r < 0.07, *p >* 0.05, all). Thus, these variables had no significant effect on our measures regarding the efficacy and success of communication, neither themselves, nor through gaze coordination.

#### 4.2.4 Prosodic coordination

Somewhat to our surprise (given the findings of previous research), no associations were found between prosodic coordination and any of the subjective or objective outcome variables. Previous studies have reported that prosodic coordination was associated with increased liking, rapport, engagement in a task, naturalness of the conversation flow and overall quality of the conversation (Lakin and Chartrand, 2003; De Looze et al., 2014; De Looze and Rauzy, 2011; Michalsky et al., 2018). However, there is also evidence for only moderate effects of prosodic coordination on long-term relationship outcome (Pardo et al., 2012), while Pérez et al. (2016) found that - in some cases - less coordination in prosody is associated with more positive outcomes (speaker engagement). In this study, even though coordination was present for pitch and intensity as well, we found neither positive, nor negative associations. It is possible that prosodic coordination might be better accounted for by DST, suggesting that alignment in acoustic features of speech might be a “byproduct” of communication and does not aid interaction in a straightforward manner. However, the current null results do not provide strong evidence for this. Further investigation is needed to better understand the effects of prosodic coordination on communication outcome.

### 4.3 Effects of manipulations

Regarding the effect of condition on the various measures of IC, we found moderate effects of the familiarity and the modality manipulations (indexed by the Random Forest models). Some previous studies have shown that pre-existing relationships induced higher levels of IC (McIntosh, 2006), whereas others reported no differences between strangers and friends, except for romantically related couples (e.g., see Djalovski et al., 2021 for movement coordination). Our results suggest that unfamiliar partners show greater IC in the three most important variables used for classification (see Supplementary Figure 5 and section *3.4 Assessing the effects of the experimental conditions on IC*) - compared to the ones who were already acquainted before the BG. It is possible that uncertainty in the unfamiliar condition prompted the partners to accommodate more to their counterpart to signal affiliation, as opposed to in a situation where a pre-existing relationship provides a sense of security for the interactants (for example in mother- vs. stranger-child dyads, Walden and Kim, 2005, for a detailed discussion of mother–child synchrony, see Bell, 2020). This is also highlighted by the fact that participants’ reports on liking were the lowest in the unfamiliar condition (shown by the significant effect of experimental condition on Liking), possibly increasing the need for IC more to compensate for the lower levels of sympathy. As for the modality manipulation, our data showed a moderate effect suggesting that the availability of unimodal information only results in weaker IC between the partners in the three most important classifier variables, compared to multimodal information (Figure 5A). This result is consistent with those of Wright et al. (2014) and Dias and Rosenblum (2016), suggesting that visual cues from the partner could have potentially acted as reinforcements for further alignment, especially for motion coordination (see also Richardson et al., 2007b for similar effects in a rocking-chair paradigm).

The variables that describe the difficulty of the BG and the elapsed time (BG_round and Time_before_BG_round) were associated with both objective and subjective outcomes (Figures 3, 4). Therefore, these results suggest that the increasing difficulty and possibly fatigue led to decreased communication efficacy, and was negatively associated with the subjective impression of communication quality, despite the fact that participants became more familiar with the game. The predictor variables BG_round and Time_before_BG_round involve multiple overlapping factors (see the increases in effect size in appendix Supplementary Table 6 from the full model including both variables to the model omitting one or the other) that include the change in game difficulty in progressive rounds, fatigue, and learning. These factors cannot be fully separated within the current data set, but results of our *post-hoc* analysis showed that the time spent in the game (see section *3.2 Predicting objective measures of communication outcome*) had an effect even when task difficulty was controlled, suggesting that at least difficulty and learning/fatigue both significantly affected the outcome variables.

However, game difficulty did not appear to modulate the level of coordination between the partners (see section *3.5. Assessing the effects of task difficulty on IC*). This suggests that the effects of game difficulty (BG_round) did not influence the outcomes through modulating IC between individuals, rather, performance was directly affected by task difficulty.

## 5 Study limitations and future directions

Data collection was affected by the COVID-19 pandemic: while the original design called for direct face-to-face communication, in order to reduce the chance of spreading the virus (in accordance with the temporary measures of the research institute), the two participants were seated in separate laboratories. As a result, the interaction of the participants was face-to-face only in virtual space. This measurement setup induced some inherent limitations on studying naturalistic human interaction. First, real eye contact between the two interlocutors was not possible due to the placement of webcams: when participant A was looking at the eye of participant B, participant B perceived that participant A was looking down at her/his screen. Second, also because of the camera placement, participants could not see exactly where their partner was looking at on their own screen. Third, as both players used their own screens and not a common screen or board, and the locations of tokens were the same for the two players, when both participants directed their gaze at the same part of their own screen, this was seen as an anti-parallel movement in the webcam’s video. This effect modelled the situation when two players are sitting facing each other in front of their own screens, as opposed to previous studies using common display items. Fourth, because of the camera placement, participants could only see each other from the shoulders up. In previous studies, communication in on-line video situations was associated with reduced turn-taking speed (Tian et al., 2024), less natural use of body cues (especially if only the head was visible (Tian et al., 2024; Zubek et al., 2022), and decreased perceived eye contact (Basch et al., 2021) compared to real-world face-to-face interactions. Averted gaze (the person in the video not looking into the camera) was found to decrease perceived empathy, perspective-taking, trustworthiness and emotional closeness (Breil and Böckler, 2021), and the lack of eye contact was associated with reduced communication efficiency, trust, and impression-formation (Bohannon et al., 2013). It was also shown that visual cues do not necessarily improve the collective intelligence of physically separated group members (Tomprou et al., 2021). Thus, the constraints associated with the on-line setup in the present study might have modulated the relationship between IC and communication outcome measures.

The experimental design confounded the effects of learning, fatigue, and increasing difficulty associated with BG rounds, as difficulty increased monotonously during the session. As a result of the increase in difficulty, participants might have had a harder time arriving at suitable negotiation outcomes for both parties, which might have introduced some competing strategies in later rounds of the game. This could have potentially limited the effect of IC on objective outcomes. Some currently available paradigms offer solutions to mitigate the effect of competitiveness by keeping difficulty levels equal throughout an experimental session (e.g., Reitter and Moore, 2014). However, the effects of fatigue and monotony (due to repetitiveness) are still a relevant factor with these types of tasks as well.

We found no significant model for the measures derived from collected wealth, indexing communication gain. However, because the task of collecting virtual money was less stressed in the instructions (it was the secondary task with collecting the must-have tokens being the primary one), participants often stopped the game soon after collecting these tokens. They might have felt that maximizing task performance also for the secondary task would have led to potential disagreements with their partner in later games. Thus, while the task had a built-in competitive element, which could have biased the participants’ strategies toward a competitive approach, the more cooperative strategy adopted by most participants (attested by often stopping once the primary goal of both participants has been achieved) may not have been optimal for the secondary task, but could have potentially served prosocial functions (Nowak and Sigmund, 2005; Gallotti et al., 2017).

We acknowledge that considering the extensive evaluation of IC in many forms of behavior (and physiology), the number of significant effects (and corresponding effect sizes) on the outcome of conversation are rather small. A previous study focusing on the core dimensions of conversation dynamics and their relationship to objective and relational outcomes in negotiation (Di Stasi et al., 2024) found that conversation measures explained 9 and 8% of the variance in objective and relational outcomes, respectively. The authors compared these effect sizes to previous findings in negotiation studies and concluded that the effect sizes are nontrivial. The effect sizes in our study are comparable to those reported by Di Stasi et al. (2024). It is possible that different forms of coordination characterize different pairs (or individual interlocutors) which might not generalize in a larger sample and thus their effects may be harder to detect. This hypothesis could be tested in future research. Finally, our analysis methods only focused on the global level of IC for each of the measured variables and did not look into dynamic changes in coordination. Admittedly, this is a simplification of a complex phenomenon, and could have potentially also contributed to the fact that some of the variables describing IC failed the validation test. In the future, we plan to look at the evolution of coordination throughout each conversation and its relation to the outcome of the interaction.

## Data Availability

The datasets presented in this study can be found in online repositories. The names of the repository/repositories and accession number(s) can be found in the article/Supplementary material.
