# Supplementary material for: Interpersonal coordination in communication: effects of alignment in multiple modalities on objective and subjective task outcomes
Source: Front Psychol. 2026 Feb 25;17:1655164. doi: 10.3389/fpsyg.2026.1655164 (PMC12975734; doi:10.3389/fpsyg.2026.1655164)
Supplement: Supplementary file 1 [file Data_Sheet_1.pdf]

Supplementary Material

1 Materials and Methods

1.1 Power analysis

The three conditions were analysed together (overall sample size 124 pairs) in a multivariate analysis using predictors selected by LASSO regularization for each outcome measure separately. The number of selected predictors varied across outcome measures, the largest model contained 8 predictors. For multiple regression with 8 predictors, medium effect size, and an alpha level of 0.05, with a sample size of 124 pairs, the estimated statistical power is 0.87. This power is sufficient for finding potential robust effects in our sample.

1.2 Procedure

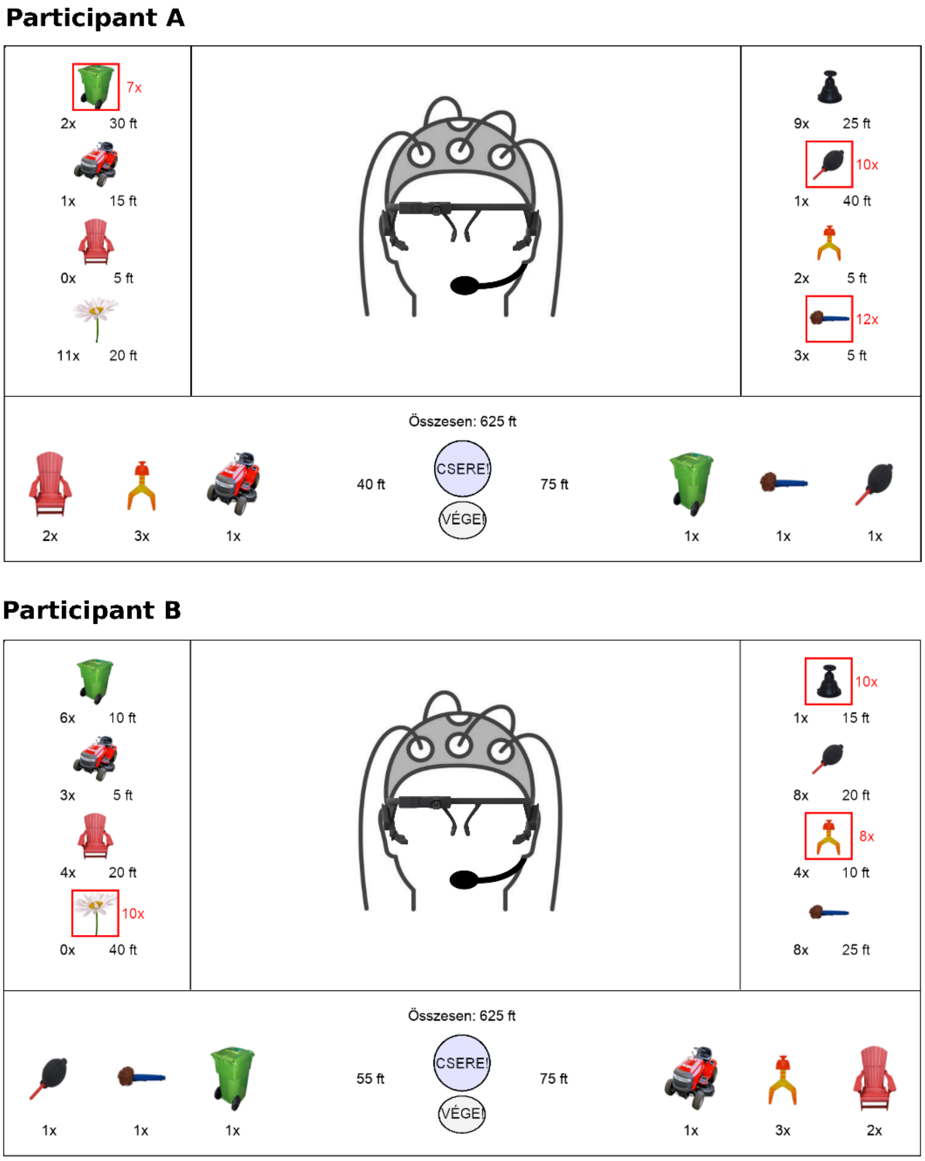

Supplementary Figure 1. **Graphical user interface (GUI) of the BG.**

GUI for participant A (top) and for participant B (bottom) captured at the same time. This scenario depicts how participants could trade tokens in a way that both of them would realize profit. See the legend of Figure 1 for details.

1.2.1 Round-to-round Increasing the Difficulty in the Bargaining Game

The potential wealth flow (the overall wealth that could be gained by the pair after trading every token for which the partner had a higher price) was significantly lower in the 3rd and 4th BG round than in the 1st and 2nd BG round (Table A1). The number of different abstract tokens included in each partner's starting set was 1, 4, 4, 7 in the 1st, 2nd, 3rd, and 4th BG round, respectively. Thus, later BG rounds carried more abstract tokens and/or more difficult configuration of token prices, allowing fewer transaction options and less profit to be collected.

Supplementary Table 1. **Increasing difficulty of BG rounds.** Potential wealth flow is denoted in arbitrary units.

BG round	Number of abstract tokens	Potential wealth flow
1	1	655
2	4	695
3	4	225
4	7	225

1.3 Data Acquisition

1.3.1 Materials

The EEG, the motion capture system, and the wearable eye tracker were connected to a data acquisition PC in each laboratory. Separate PCs were responsible for inter-laboratory communication and audiovisual recordings. Audio was transferred between the two laboratories via microphone cables, video frames via USB, and in-game actions via low-latency User Datagram Protocol (UDP) streams (Fig. A2).

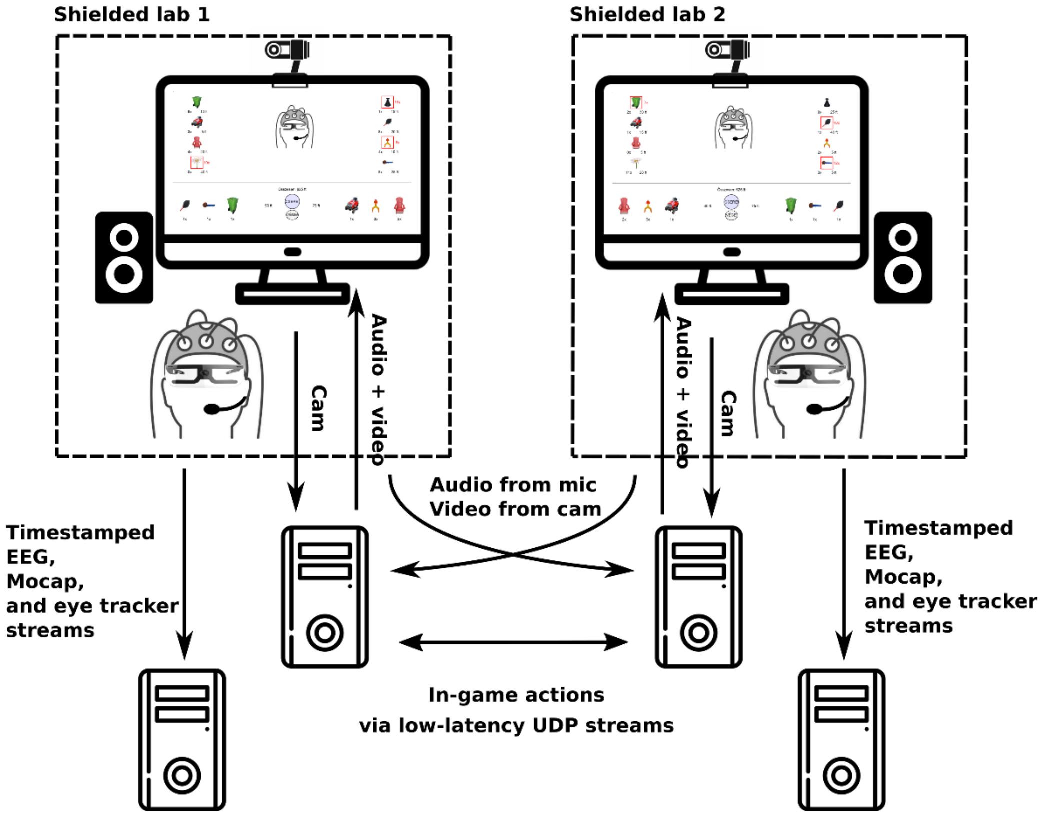

Supplementary Figure 2. **Recording signals and connections between the two laboratories.**

1.3.2 Eye Tracking

The Pupil Capture software was used to read the video streams coming from the two cameras of the eye tracker and to calculate pupil size and gaze position based on these video streams. Zero gain, auto exposure aperture priority mode, and auto white balance temperature were used. The reliability of gaze tracking was quantified for a subset of participants (176 individuals) using the built-in functionality of the Pupil Capture software to measure the accuracy (the average angular offset between fixation locations and the corresponding locations of the fixation targets) and angular precision (the root mean square of the angular distance between successive samples during fixation) of the gaze tracking. The mean value and standard deviation of accuracy was 2.78 ± 0.49 degrees of visual angle, the mean value and standard deviation of precision was 0.19 ± 0.04 degrees of visual angle.

1.4 Communication Outcome Measures

1.4.1 Subjective Measures of Communication Outcome

Supplementary Table 2. **Hungarian version of the questionnaire items used as subjective communication outcome measures.**

Name	Text of the questionnaire items in Hungarian
Perceived_Difficulty	“Nehéznek éreztem az előző feladatot.”
Naturalness_of_Conversation	“Folytonosnak, természetesnek éreztem az előző beszélgetést.”
Rapport	“Megtaláltuk a közös hangot a társammal az előző feladat során.”
Liking	“Kedvelem a társamat.”
Disagreement	“Gyakran fordult elő, hogy nem értettem egyet a társammal az előző feladat során.”
Perceived_Synchrony	“Úgy érzem, hogy összhangban voltunk a társammal az előző feladat során.”
Difference_in_Leading	“Én irányítottam a beszélgetést az előző feladat során.”
Pleasantness_of_the_Task	“Kellemes/élvezetes volt az előző feladat.”
Surprisal_of_Behavior	“Meglepő volt a társam viselkedése az előző feladat során.”
Truthfulness	“Mindig igazat mondtam az előző feladat során.”
Individual_Task_Performance	“Úgy gondolom, hogy nekem volt nagyobb nyereségem az előző játékban.”
Efficiency_in_Trading	“Minden lehetőséget kihasználtunk a cserére a társammal.”
Predictability_of_the_Partner	"A társam kiszámíthatóan játszotta a játékot."

1.5 Measures Assessing IC

1.5.1 Pupil Size Coordination

Pupil diameter and gaze position were estimated using the automatic 3D pupil detection and gaze mapping algorithms implemented in the Pupil Capture software. We opted for the 3D model as it offers robust pupil detection and compensates for slippage with the disadvantage of lower gaze position accuracy (recommended by Pupil Labs). First, data points where confidence values of pupil detection obtained from the Pupil Capture software were not within the acceptable range (above 0.6) were considered missing. Another filter removed pupil data which fell outside the physiologically feasible range (1.5-9 mm based on Mathôt, 2018) and/or was 3 standard deviations above or below the median pupil diameter.

Certain artifacts (‘spikes’) caused by blinks can remain in the signal after removing invalid samples based on pupil confidence thresholds (given by the Pupil Capture software). These artifacts are characterized by disproportionately large changes in pupil size – or dilation speeds. Following the guidelines of Kret & Sjak-Shie (2019), spikes were removed using a filter based on the median absolute deviation of the speed of diameter change between successive samples. We used the following formula, where d[i] denotes the pupil size with the

corresponding timestamps t[i], and the dilation speed at each sample d'[i] is then calculated as the maximum absolute change relative to either the preceding or the succeeding sample:

$$d'[i] = max(| (d[i] - d[i-1]) / (t[i] - t[i-1]) | , | (d[i+1] - d[i]) / (t[i+1] - t[i]) |)$$

Dilation speed outliers were detected using the median absolute deviation (MAD) value, which was calculated from the dilation speed series, and then multiplied by a constant (n) and summed with the median dilation speed:

$$MAD = median(| d'[i] - median(d') |),$$

$$threshold = median(d') + n * MAD$$

The constant was set to 3 based on visual examination of the artifacts. The dilation speed filtering method was applied twice to remove any additional dilation speed outliers missed by the first filter (due to for example the clustering of 2 or more outlier samples).

To deal with the remaining noise in the signal before interpolation, we applied an additional filter which removed small clusters of spurious samples ('loners') that were temporally separated from other valid samples. Clusters were labeled 'loners' and were set to invalid if they were surrounded by invalid samples (resulting from previous filtering steps) and had a maximum length of 2 samples. This threshold was chosen as it yielded the best results for removing interpolation spikes (determined by visually inspecting the data). Then, the missing pupil diameter data were linearly interpolated and low-pass filtered (a fifth-order Butterworth low-pass filter with 10 Hz cutoff frequency using two-direction zero-phase filtering).

Finally, the effect of brightness on pupil diameter values was regressed out. Brightness was estimated by applying a circular mask to video frames of the world camera corresponding to the central 15 degrees of the visual field, and calculating the average brightness values within the circular mask. The size of the circular mask was chosen based on the study of Bradley and colleagues (Bradley et al., 2017), and after testing different circular masks corresponding to the central 6-19 degrees of the visual field. A linear regression model was fitted to the estimated brightness values and the corresponding pupil diameter values. As the pupil reacts to brightness changes with a short delay (Bergamin & Kardon, 2003; Tsujimura & Tokuda, 2011; Fotiou et al., 2007), the linear regression model was fitted considering a delay between brightness values and the corresponding pupil diameter values. Different delays between 0 and 5 s were tested, a delay of 0.5 s yielded the highest model fit, and therefore, this value was used. In the subsequent analyses, residual pupil diameter values (not explained by the linear regression model) were used. No other possible confounding factors were removed. Before estimating pupil size coordination using dynamic time warping, pupil diameter data was resampled at 30 Hz.

1.5.2 Gaze Coordination

While the game-related areas were fixed rectangles on the screen, the participant's face was detected using the OpenFace toolkit (Baltrušaitis et al., 2016). Fixations were detected using the built-in fixation detector of the Pupil Player software (data analysis software provided by Pupil Labs) applying a dispersion-based algorithm. The maximum dispersion threshold was set to 2 degrees, the minimum fixation duration threshold was set to 60 ms. These thresholds allowed merging fixations that were separated only by microsaccades (Drews & Dierkes, 2024).

To test the effect of fixation detection on the calculated gaze coordination ratio, gaze coordination was also estimated using a more conservative minimum fixation duration threshold (100 ms) and with no fixation detection (gaze coordination was calculated based on whether the two participants were looking at the same AOI at the same time, regardless of if any of them were fixating). Results calculated with these alternative fixation detection approaches are reported in the Results section of this Appendix (*2.2 Effect of Gaze Coordination on Outcome Variables with Alternative Fixation Detection Approaches*).

1.5.3 Motion Coordination

First, head position data were band-pass filtered between 0.01 - 30 Hz to remove slow drift and high-frequency artifacts. To calculate the squared velocity, the derivative of position with respect to time was computed and squared separately along the x, y, z directions. Finally, squared velocities along the x, y, z directions were summed and integrated within the sliding window, and the time series of integrated values were correlated between the two participants by Pearson's correlation coefficient. Fujiwara and Daibo recommended the frequency range of 0-4 Hz for head movement coherence analysis in unstructured face-to-face conversation (Fujiwara & Daibo, 2016). Tschacher et al. investigated nonverbal synchrony in dyadic interactions using motion energy analysis and cross correlation, and suggested the consideration of time lags up to 5 s (both positive and negative) (Tschacher et al., 2014; Ramseyer & Tschacher, 2011). We used a window size of 6 s and a window step size of 8.33 ms (1 sample).

1.5.4 Audio Preprocessing

Missing segments in the audio stream (buffer underflow events during streaming) were filled with silence and deviations from the nominal sampling rate were corrected with resampling. Although the experimental rooms were sound-attenuated, a minimal noise reduction was applied to eliminate both cross-talk (other participant's speech played from the speaker which is picked up by the microphones) and the occasional line noise.

1.5.5 Correlations – Separately for the Outcome and the Predictor Variables

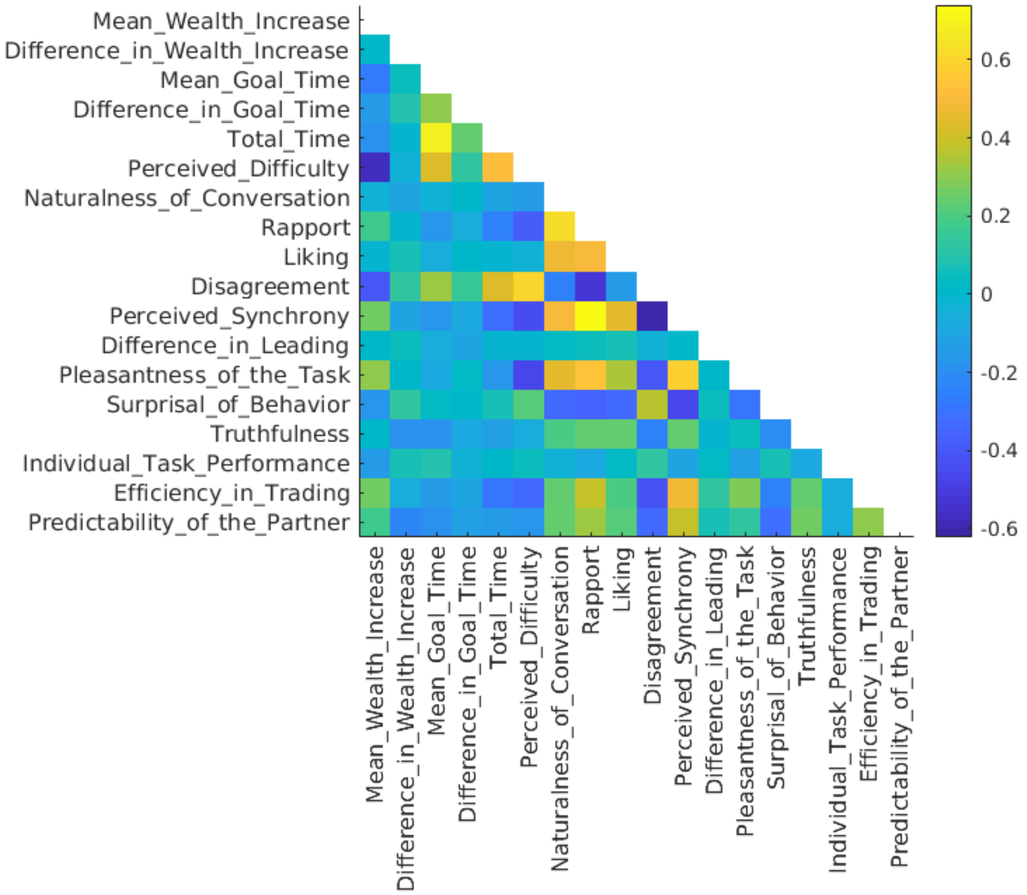

Supplementary Figure 3. **Correlation between communication outcome measures.**

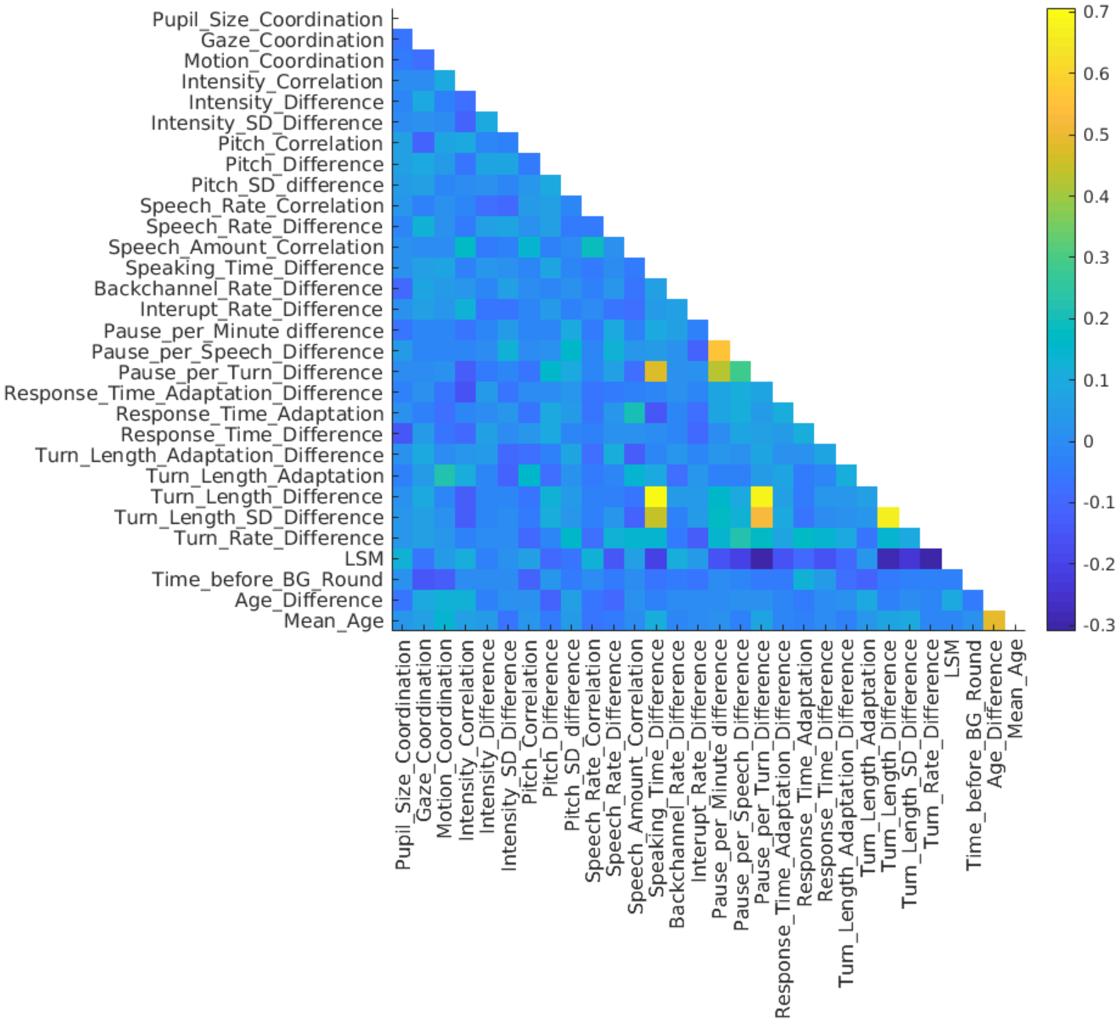

Supplementary Figure 4. **Correlation between predictor variables of communication outcome (excluding categorical variables).**

1.6 Statistical Analysis

Supplementary Table 3. **The number of pairs with missing predictor variable data in each Condition and BG Round.** Each column represents a condition - BG round combination: *condition-abbreviation_BG-round,* where. BL is Baseline condition, UF is Unfamiliar condition, and UM is Unimodal condition.

	BL_1	BL_2	BL_3	BL_4	UF_1	UF_2	UF_3	UF_4	UM_1	UM_2	UM_3	UM_4
Pupil_Size_Coordination	9	8	8	6	10	7	7	4	10	7	7	5
Gaze_Coordination	0	0	0	1	1	0	0	0	1	0	1	0
Motion_Coordination	2	2	2	2	2	1	2	0	2	1	0	1
Intensity_Correlation	0	2	1	0	1	1	1	1	0	2	0	0
Intensity_Difference	0	0	0	0	0	0	0	1	0	0	0	0
Intensity_SD_Difference	0	0	0	0	0	0	0	1	0	0	0	0

Pitch_Correlation	0	2	1	0	1	1	1	1	0	2	0	0
Pitch_Difference	0	0	0	0	0	0	0	1	0	0	0	0
Pitch_SD_Difference	0	0	0	0	0	0	0	1	0	0	0	0
Speech_Rate_Correlation	0	2	1	0	1	1	1	1	0	2	0	0
Speech_Rate_Difference	0	0	0	0	0	0	0	1	0	0	0	0
Speech_Amount_Correlation	0	2	1	0	1	1	1	1	0	2	0	0
Speaking_Time_Difference	0	0	0	0	0	0	0	1	0	0	0	0
Backchannel_Rate_Difference	0	0	0	0	0	0	0	1	0	0	0	0
Interrupt_Rate_Difference	0	0	0	0	0	0	0	1	0	0	0	0
Pause_per_Minute _Difference	0	0	0	0	0	0	0	1	0	0	0	0
Pause_per_Speech_Difference	0	0	0	0	0	0	0	1	0	0	0	0
Pause_per_Turn_Difference	0	0	0	0	0	0	0	1	0	0	0	0
Response_Time_Adaptation _Difference	0	0	0	0	0	0	0	1	0	0	0	0
Response_Time_Adaptation	0	0	0	0	0	0	0	1	0	0	0	0
Response_Time_Difference	0	0	0	0	0	0	0	1	0	0	0	0
Turn_Length_Adaptation _Difference	0	0	0	0	0	0	0	1	0	0	0	0
Turn_Length_Adaptation	0	0	0	0	0	0	0	1	0	0	0	0
Turn_Length_Difference	0	0	0	0	0	0	0	1	0	0	0	0
Turn_Length_SD_Difference	0	0	0	0	0	0	0	1	0	0	0	0
Turn_Rate_Difference	0	0	0	0	0	0	0	1	0	0	0	0
LSM	0	0	0	1	0	0	0	1	0	0	0	1
BG_Round	0	0	0	0	0	0	0	0	0	0	0	0
Condition	0	0	0	0	0	0	0	0	0	0	0	0
Time_before_BG_Round	0	0	0	0	0	0	0	0	0	0	0	0
Age_Difference	0	0	0	0	0	0	0	0	0	0	0	0
Mean_Age	0	0	0	0	0	0	0	0	0	0	0	0
Gender	0	0	0	0	0	0	0	0	0	0	0	0
Handedness	0	0	0	0	0	0	0	0	0	0	0	0

2 Results

2.1 Permutation tests - Real and Pseudo Pair Comparisons

Supplementary Table 4. **IC variables with non-significant differences between real and pseudo pairs.** For variables, where a transformation was applied before statistical analysis, the upper value shows the original mean and standard deviation (SD), the lower value shows the transformed mean and SD.

Variable Name	Mean (SD) real	Mean (SD) pseudo
Intensity_Difference	3.801 (2.752)<br>1.023 (0.951)	4.189 (3.173)<br>1.103 (0.967)
Pitch_Difference	18.883 (14.452)<br>2.527 (1.103)	20.137 (15.182)<br>2.614 (1.060)
Pitch_SD_Difference	10.675 (9.111)<br>2.003 (0.965)	10.473 (8.340)<br>1.960 (1.038)
Speech_Rate_Difference	0.558 (0.479)<br>-0.651 (0.703)	0.572 (0.484)<br>-0.635 (0.718)
Speaking_Time_Difference	8.148 (6.682)<br>1.691 (1.065)	8.890 (6.904)<br>1.805 (1.039)
Backchannel_Rate_Difference	0.127 (0.130)<br>-1.627 (0.531)	0.127 (0.125)<br>-1.612 (0.505)
Pause_per_Minute _Difference	5.091 (3.777)<br>1.296 (0.985)	5.090 (4.017)<br>1.269 (0.998)
Pause_per_Speech_Difference	0.085 (0.066)<br>-1.744 (0.340)	0.089 (0.072)<br>-1.731 (0.348)
Pause_per_Turn_Difference	0.269 (0.237)<br>-1.181 (0.606)	0.249 (0.212)<br>-1.213 (0.564)
Response_Time_Adaptation_Difference	0.169 (0.144)<br>-1.439 (0.499)	0.174 (0.139)<br>-1.411 (0.483)
Response_Time_Adaptation	-0.004 (0.113)	-0.002 (0.111)
Turn_Length_Adaptation _Difference	0.156 (0.127)<br>-1.474 (0.463)	0.159 (0.124)<br>-1.456 (0.459)
Turn_Length_Adaptation	0.025 (0.101)	0.030 (0.103)
Turn_Length_Difference	0.750 (0.650)<br>-0.448 (0.787)	0.733 (0.616)<br>-0.459 (0.780)
Turn_Length_SD_Difference	0.682 (0.558)<br>-0.511 (0.764)	0.715 (0.573)<br>-0.466 (0.763)

2.2 Effect of Gaze Coordination on Outcome Variables with Alternative Fixation Detection Approaches

Applying a minimum fixation duration threshold of 100 ms and the maximum dispersion threshold of 2 degrees, Gaze_Coordination was a significant predictor for two communication efficacy measures: Mean_Goal_Time ($p<0.01$, $f^2=0.033$), and Total_Time ($p<0.001$,

f^2=0.069), and one subjective communication outcome: Perceived_Difficulty ($p<0.001$, f^2=0.033).

Applying no fixation detection, Gaze_Coordination was a significant predictor for all three communication efficacy measures: Mean_Goal_Time ($p<0.001$, f^2=0.047), Difference_in_Goal_Time ($p<0.01$, f^2=0.021) and Total_Time ($p<0.001$, f^2=0.110), and one subjective communication outcome: Perceived_Difficulty ($p<0.0001$, f^2=0.043).

2.3 Summary of LMM Results

Supplementary Table 5. **LMM results for objective measures of communication efficacy and significant predictor variables.**

Output variable	Model fit (R^2)	Input variable	Effect size (f^2)	Direction	*p*-value
Mean_Goal_Time	0.152	Time_Before_BG_Round	0.114	Pos.	<0.0001
		Gaze_Coordination	0.038	Neg.	<0.01
Difference_in_Goal_Time	0.018	Gaze_Coordination	0.018	Neg.	<0.05
Total_Time	0.164	Time_Before_BG_Round	0.055	Pos.	<0.0001
		Gaze_Coordination	0.087	Neg.	<0.0001

Supplementary Table 6. **LMM results for subjective communication outcomes and significant predictor variables.** Effect size was generally calculated based on the original full model. For BG_Round and Time_Before_BG_Round, two effect sizes can be shown. The second effect size is shown only when both variables survived the LASSO selection, and the given variable was a significant contributor to the full model. In these cases, the second effect size (in parenthesis) was calculated based on a model excluding BG_Round for Time_Before_BG_Round and excluding Time_Before_BG_Round for BG_Round.

Output variable	Model fit (R^2)	Input variable	Effect size (f^2)	Direction	*p*-value
Perceived_Difficulty	0.465	BG_Round	0.051 (0.759)	Pos.	<0.0001
		Time_Before _BG_Round	0.017 (0.703)	Pos.	<0.05
		Gaze_Coordination	0.037	Neg.	<0.001
Naturalness_of_Conversation	0.055	Age_Difference	0.027	Pos.	<0.05
		Interrupt_Rate_Difference	0.012	Pos.	<0.05

		LSM	0.019	Pos.	<0.01
Rapport	0.065	Time_Before_BG_Round	0.070	Neg.	<0.0001
Liking	0.182	Condition	0.086	Neg.	<0.001
		Gender	0.096	Neg.	<0.001
		Motion_Coordination	0.005	Pos.	<0.05
		Turn_Rate_Difference	0.007	Neg.	<0.05
Disagreement	0.227	BG_Round	0.003 (0.283)	Pos.	<0.001
Pleasantness_of_the_Task	0.140	BG_Round	0.003 (0.096)	Neg.	<0.05
Surprisal_of_Behavior	0.084	BG_Round	0.028	Pos.	<0.001
		Gender	0.038	Pos.	<0.05
		Turn_Rate_Difference	0.022	Pos.	<0.01
Efficiency_in_Trading	0.159	Time_Before_BG_Round	0.156	Neg.	<0.0001
		Interrupt_Rate_Difference	0.013	Pos.	<0.05
		LSM	0.014	Pos.	<0.05
Predictability_of_the_Partner	0.054	BG_Round	0.020	Neg.	<0.001

2.4 Post-hoc Analysis of the Effects of Gaze Coordination on Subjective Outcomes

One may assume that the effect of Gaze_Coordination on Perceived_Difficulty was due to Gaze_Coordination varying together with the length of the game; i.e., shorter total game time leading to perceiving the game as easier. We ran a post-hoc analysis including the objective outcomes as potential predictors in a LMM analysis and found that only Total_Time emerged as a significant predictor in this new model, Gaze_Coordination did not ($p<0.001$, $f2=0.054$). This suggests that higher Gaze_Coordination resulted in shorter games, which in turn felt easier for the players. Unfortunately running a proper mediation analysis was not possible in this data set due to its limited size (Fritz & MacKinnon, 2007).

2.5 Post-hoc Analysis of the Effects of Conversation Structure Coordination

Supplementary Table 7. **Contingency table for the relationship between individual interrupt rate values, and individual questionnaire ratings of Naturalness_of_Conversation within the pairs.** P1 denotes participant 1, P2 denotes participant 2. Cells of the table show observed frequencies of the combinations when P1 or P2 had higher or equal interrupt rates and gave higher, lower or equal scores for the questionnaire item Naturalness_of_Conversation.

	**Interrupt Rate**			
**Naturalness_of_Conversation**	P1 > P2	P1 = P2	P1 < P2	Sum
P1 > P2	69	35	77	181
P1 = P2	45	18	48	111
P1 < P2	75	26	59	160
Sum	189	79	184	452

χ^2=3.11, degrees of freedom=4, p>0.1

2.6 Variable Importance Analysis for the Random Forest Model on BG Difficulty

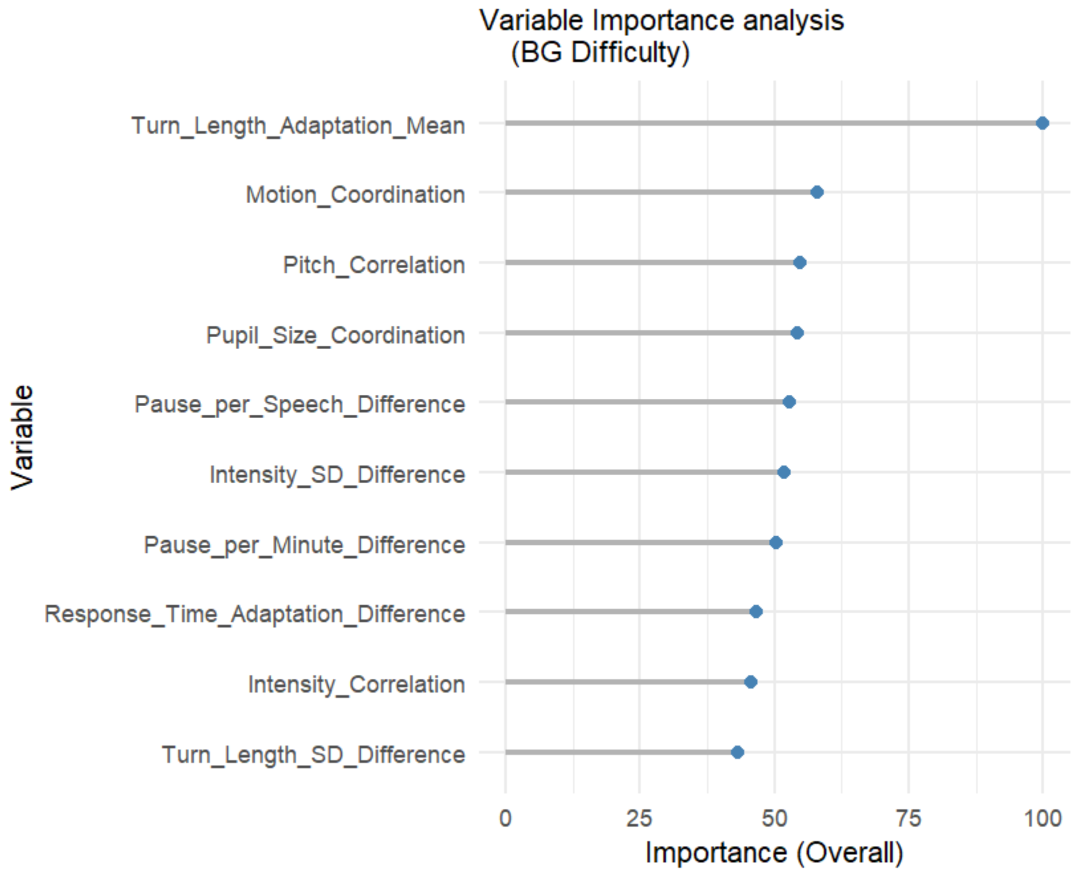

Supplementary Figure 5. **Variable Importance analysis from the Random Forest model on BG difficulty.** Overall importance is depicted on a relative scale from 0-100, where 100 is

assigned to the most important variable, followed by other variables in proportion to the top variable. The first 10 (out of 27) variables are shown.

Data availability statement

The dataset presented in this study excluding sensitive data (raw audio recordings and transcripts) is available at *https://repo.researchdata.hu/dataset.xhtml?persistentId=hdl:21.15109/ARP/0BDBAT*

References

Baltrušaitis, T., Robinson, P., & Morency, L. P. (2016, March). Openface: an open source facial behavior analysis toolkit. In 2016 IEEE winter conference on applications of computer vision (WACV) (pp. 1-10). IEEE. https://doi.org/10.1109/wacv.2016.7477553

Bergamin, O., & Kardon, R. H. (2003). Latency of the pupil light reflex: sample rate, stimulus intensity, and variation in normal subjects. *Investigative Ophthalmology & Visual Science, 44*(4), 1546-1554. https://doi.org/10.1167/iovs.02-0468

Bradley, M. M., Sapigao, R. G., & Lang, P. J. (2017). Sympathetic ANS modulation of pupil diameter in emotional scene perception: Effects of hedonic content, brightness, and contrast. *Psychophysiology, 54*(10), 1419-1435. https://doi.org/10.1111/psyp.12890

Drews, M., & Dierkes, K. (2024). Strategies for enhancing automatic fixation detection in head-mounted eye tracking. *Behavior Research Methods, 56*(6), 6276-6298. https://doi.org/10.3758/s13428-024-02360-0

Fotiou, D. F., Brozou, C. G., Tsiptsios, D. J., Fotiou, A., Kabitsi, A., Nakou, M., ... & Goula, A. (2007). Effect of age on pupillary light reflex: evaluation of pupil mobility for clinical practice and research. *Electromyography and clinical neurophysiology, 47*(1), 11.

Fritz, M. S., & MacKinnon, D. P. (2007). Required sample size to detect the mediated effect. Psychological science, 18(3), 233-239. https://doi.org/10.1111/j.1467-9280.2007.01882.x

Kret, M. E., & Sjak-Shie, E. E. (2019). Preprocessing pupil size data: Guidelines and code. Behavior research methods, 51, 1336-1342. https://doi.org/10.3758/s13428-018-1075-y

Mathôt, S. (2018). Pupillometry: Psychology, physiology, and function. *Journal of cognition, 1*(1). https://doi.org/10.5334/joc.18

Tsujimura, S. I., & Tokuda, Y. (2011). Delayed response of human melanopsin retinal ganglion cells on the pupillary light reflex. *Ophthalmic and Physiological Optics, 31*(5), 469-479. https://doi.org/10.1111/j.1475-1313.2011.00846.x
